# Surfactant protein C mutation links postnatal type 2 cell dysfunction to adult disease

**DOI:** 10.1172/jci.insight.142501

**Published:** 2021-07-22

**Authors:** Sneha Sitaraman, Emily P. Martin, Cheng-Lun Na, Shuyang Zhao, Jenna Green, Hitesh Deshmukh, Anne-Karina T. Perl, James P. Bridges, Yan Xu, Timothy E. Weaver

**Affiliations:** 1 Divisions of Neonatology and Pulmonary Biology, and; 2 Division of Biomedical Informatics, Cincinnati Children’s Hospital Medical Center, Cincinnati, Ohio, USA.; 3 Department of Pediatrics, University of Cincinnati College of Medicine, Cincinnati, Ohio, USA.; 4 Division of Pulmonary, Critical Care and Sleep Medicine, National Jewish Health, Denver, Colorado, USA.

**Keywords:** Cell Biology, Pulmonology, Cell stress, Protein misfolding, Pulmonary surfactants

## Abstract

Mutations in the gene *SFTPC*, encoding surfactant protein C (SP-C), are associated with interstitial lung disease in children and adults. To assess the natural history of disease, we knocked in a familial, disease-associated *SFTPC* mutation, L188Q (L184Q [LQ] in mice), into the mouse *Sftpc* locus. Translation of the mutant proprotein, proSP-C^LQ^, exceeded that of proSP-C^WT^ in neonatal alveolar type 2 epithelial cells (AT2 cells) and was associated with transient activation of oxidative stress and apoptosis, leading to impaired expansion of AT2 cells during postnatal alveolarization. Differentiation of AT2 to AT1 cells was also inhibited in ex vivo organoid culture of AT2 cells isolated from LQ mice; importantly, treatment with antioxidant promoted alveolar differentiation. Upon completion of alveolarization, *Sftpc^LQ^* expression was downregulated, leading to resolution of chronic stress responses; however, the failure to restore AT2 cell numbers resulted in a permanent loss of AT2 cells that was linked to decreased regenerative capacity in the adult lung. Collectively, these data support the hypothesis that susceptibility to disease in adult LQ mice is established during postnatal lung development, and they provide a potential explanation for the delayed onset of disease in patients with familial pulmonary fibrosis.

## Introduction

Alveolar type 2 epithelial cells (AT2 cells) synthesize and secrete pulmonary surfactant, which is essential for respiration. Surfactant is a complex mixture of lipids and proteins that forms a bioactive film at the alveolar air-liquid interface and reduces surface tension at end expiration. The protein components of surfactant, particularly the hydrophobic peptides surfactant protein B (SP-B) and SP-C, promote the formation and maintenance of a stable surfactant film (1). Expression of SP-B is absolutely required for adaptation to air breathing at birth and postnatal survival, whereas SP-C is dispensable for postnatal lung function. However, although the role of SP-C in surfactant homeostasis is not well understood, mutations in the gene encoding SP-C (*SFTPC* in humans; *Sftpc* in mice) have been linked to development of interstitial lung disease.

Human SP-C is synthesized as a 197–amino acid integral membrane precursor protein consisting of a cytosolic N-terminal domain (residues 1–23) that directs intracellular trafficking of the proprotein, a hydrophobic membrane–spanning domain that comprises most of the biophysically active, secreted mature peptide (residues 24–58), and a luminal linker region (residues 59–89), followed by the C-terminal BRICHOS domain (residues 90–197). The luminal BRICHOS domain functions as an intramolecular chaperone that promotes proper folding and insertion of the transmembrane α-helix (2, 3). The SP-C proprotein (proSP-C) traverses the secretory pathway from the endoplasmic reticulum (ER) to the multivesicular body (MVB)/late endosome, where Nedd4-2–mediated ubiquitination of a PY motif in the cytosolic domain allows for internalization of proSP-C from the limiting membrane of the MVB to internal vesicles, followed by proteolytic removal of the N- and C-terminal domains to generate the mature SP-C peptide (4, 5). Subsequent fusion of the MVB with a lamellar body (LB) leads to SP-B–mediated incorporation of vesicles and SP-C into surfactant membranes (lamellae) of the LB. The fully assembled lipid-protein surfactant complex is stored in the LB until its contents are released into the alveolar airspaces via exocytosis. *SFTPC* mutations leading to degradation or mistrafficking of proSP-C are linked to pathogenesis in humans.

Monoallelic mutations in the *SFTPC* gene are associated with development of diffuse parenchymal lung disease in both children and adults (6). Significant variability exists in the age of onset and clinical presentation of disease, which can range from nonspecific interstitial pneumonitis in infancy (7) to fulminant pulmonary fibrosis in adults (8). Adults with *SFTPC* mutations develop fibrotic lung disease as early as 30 years of age, significantly earlier than patients with nonfamilial disease. Mutations mapping to the BRICHOS domain result in formation of a misfolded proprotein leading to ER retention, accumulation of toxic, misfolded proSP-C, and activation of the ER stress and unfolded protein response (UPR) pathways (9, 10).

The exon 5+128 T→A BRICHOS mutation, resulting in substitution of a highly conserved leucine (L) by glutamine (Q) at position 188, is one of the most well-characterized familial *SFTPC* mutations (11, 12). The L188Q mutation was identified in a large kindred of over 300 members with a long history of lung disease and is inherited in an autosomal dominant mode with incomplete penetrance and expressivity (13, 14). Several children in the kindred presented with pneumonitis associated with viral infections at the time of diagnosis, whereas adults presented with usual interstitial pneumonia, the histological correlate of idiopathic pulmonary fibrosis. In adult transgenic mice, doxycycline-induced expression of L188Q resulted in activation of ER stress and augmented lung injury associated with AT2 cell apoptosis following bleomycin challenge (15). L188Q transgenic mice that were also deficient for *Chop* were protected against bleomycin-induced injury, supporting the hypothesis that AT2 cell loss is associated with dysregulated lung repair (16). Stress responses observed in transgenic mice were consistent with acute exacerbations associated with sudden activation of transgene expression and an increase in proSP-C levels. Consistent with this hypothesis, transgenic overexpression of *Sftpc*^Δexon4^ (9) or tamoxifen-mediated increase in *Sftpc*^C121G^ expression (17) resulted in rapid induction of ER stress, apoptosis of AT2 cells, and/or lethality associated with inefficient epithelial regeneration. The severity of the phenotype correlated with expression levels of the mutant *Sftpc* allele, supporting the hypothesis that misfolded proSP-C exerts a dose-dependent, cytotoxic effect on AT2 cells.

*SFTPC* mutations are not typically associated with postnatal lethality, and symptomatic disease in infants/children may be relieved following therapeutic intervention. Since the majority of mouse models overexpressed or induced acute expression of mutant *Sftpc*, the observed phenotypes likely model the phase of disease typified by acute exacerbations. Importantly, ER stress pathways were not activated in cell lines, with constitutive expression of BRICHOS domain mutations suggesting that cells adapted to chronic stress imposed by misfolded proSP-C (18, 19). In this study, we generated a knock-in mouse model of the L188Q mutation (L184Q in mice) to follow the natural history of disease associated with mutant proSP-C expression during postnatal alveolarization. We demonstrate that cell stress, arising from a transient neonatal increase in proSP-C levels, impairs postnatal AT2 cell endowment. Upon completion of alveolarization, *Sftpc* expression is downregulated, leading to resolution of stress responses; however, the postnatal loss of AT2 cells significantly compromises the regenerative capacity of the adult lung.

## Results

### Constitutive expression of the L184Q allele is associated with impaired lung repair following injury.

A mouse cDNA construct encoding the equivalent of the human exon 5+128 T→A (L188Q) mutation was knocked into the mouse *Sftpc* locus in-frame with exon 1, resulting in an amino acid substitution at position 184 (L184Q, LQ) of the mouse proSP-C; mouse proSP-C is 4 amino acids shorter than human proSP-C (Supplemental Figure 1A; supplemental material available online with this article; https://doi.org/10.1172/jci.insight.142501DS1). The floxed neomycin (neo) resistance cassette was excised by breeding mice carrying the targeted allele to an *EIIA*-Cre transgenic line, resulting in germline deletion of the cassette (Supplemental Figure 1A). No significant changes were observed in *Sftpc* gene expression in LQ mice with or without the neo-resistance cassette, and all experiments were performed with neo-free mice.

Gross lung structure was unremarkable in mice homozygous for the LQ allele (LQ/LQ) up to 18 months of age in the absence of an exogenous stressor (Supplemental Figure 1B), suggesting that constitutive expression of the LQ allele was insufficient to initiate a pathological response. To determine if expression of the LQ allele impacted the repair capacity of the adult lung, 10- to 12-week-old WT and LQ/LQ mice were injured with the fibroproliferative agent bleomycin. Both WT and LQ/LQ mice demonstrated dose-dependent responses to single bleomycin challenge, with an augmented response in LQ/LQ mice compared with WT mice (Supplemental Figure 2A). A single high dose of 3 U/kg bleomycin resulted in mortality in LQ/LQ but not WT mice: 67% of LQ/LQ mice (4 of 6) died in the first 35 days of the study (Supplemental Figure 2B). Six months after bleomycin challenge, WT mice demonstrated variable lung repair ranging from nearly normal lung structure to sustained lung injury; in contrast, surviving LQ/LQ mice demonstrated a 2-fold increase in lung injury compared with WT (Supplemental Figure 2, C and D).

**Figure 1:**
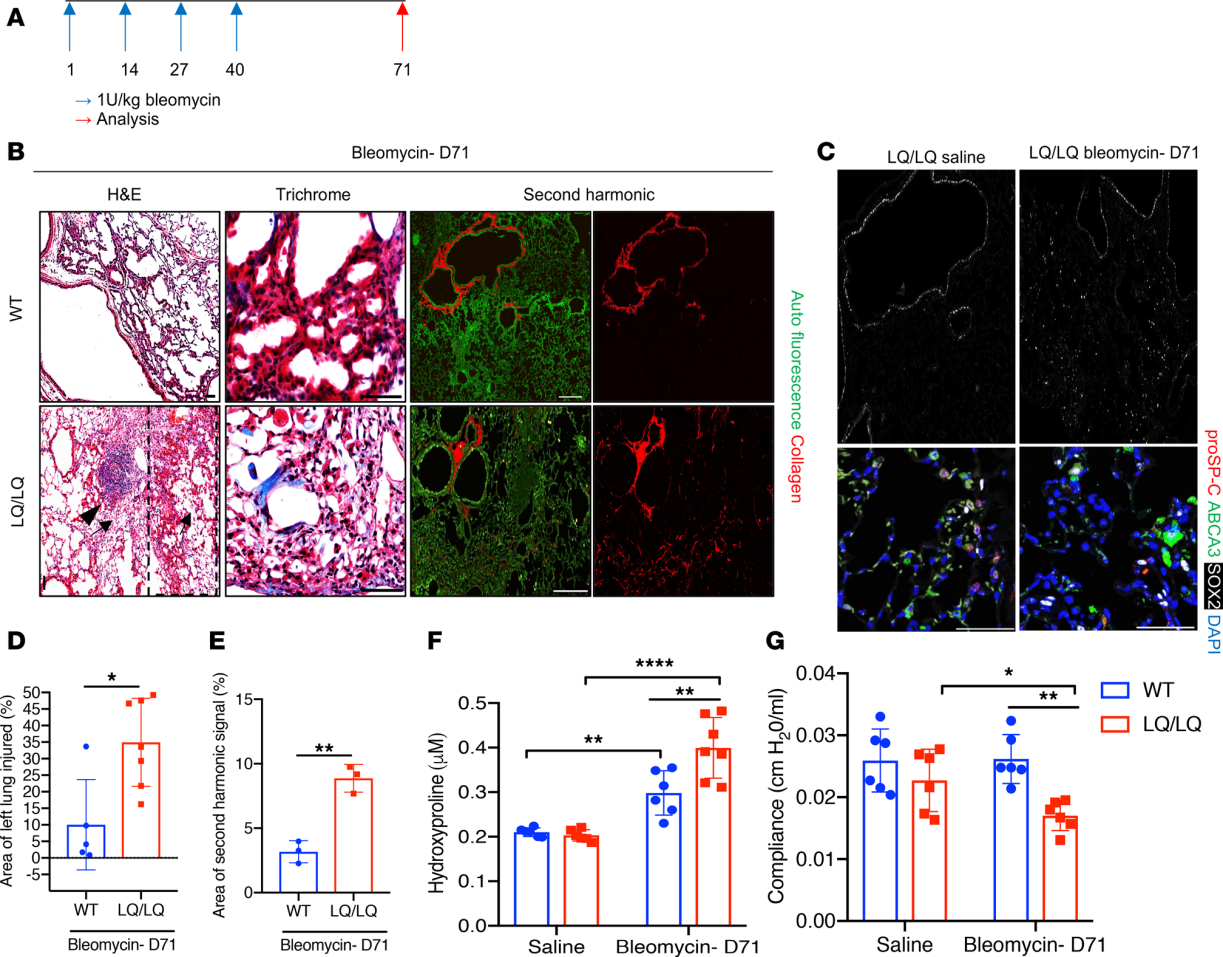
Augmented lung injury and parenchymal fibrosis in adult LQ/LQ mice. (**A**) Schematic of repetitive bleomycin challenge. (**B**) Representative 10× H&E-stained, 20× trichrome-stained, and 10× second harmonic images of left lung sections. H&E image for LQ/LQ mouse shows injured area with inflammation (arrow) including lymphocytic aggregate (arrowhead) and proximalization of distal airspaces (boxed region), juxtaposed to normal lung parenchyma. Note parenchymal collagen in trichrome (in blue) and second harmonic images (in red). Scale bars: 50 μm (H&E, trichrome) and 500 μm (second harmonic image). (**C**) Representative 10× tile scans (black and white image) and maximum intensity projection of LQ/LQ lung sections stained with proSP-C, ABCA3, and proximal epithelial cell transcription factor SOX2. Scale bar: 50 μm. (**D**) Histological score for area of left lung injured in response to bleomycin challenge on day 71 of the study. Area of injury was normalized to total area of the lung lobe. (**E**) Area of second harmonic signal (representing collagen) scored from images of 5 μm–thick paraffin sections obtained from bleomycin-challenged mice on day 71 of the study. Area of collagen signal was normalized to area of the lung parenchyma imaged with auto fluorescence of the section. For **D** and **E**, *P < 0.05, **P < 0.01 by unpaired 2-tailed *t* test. (**F**) Hydroxyproline concentration obtained from the right middle lung lobe. (**G**) Lung compliance measured by FlexiVent. For **F** and **G**, *P < 0.05, **P < 0.01, ****P < 0.0001 by 2-way ANOVA with Sidak’s multiple comparison test.

To assess lung repair following chronic injury, 10- to 12-week-old WT and LQ/LQ mice were challenged with a low dose of bleomycin (1 U/kg) once every 2 weeks for 8 weeks and were analyzed 1 month after the fourth and final dose (day 71; Figure 1A). On day 71 of the study, bleomycin-challenged LQ/LQ mice demonstrated significant lung injury marked by inflammation (Figure 1B, arrows, and Supplemental Figure 3A), lymphocytic aggregates (Figure 1B, arrowhead), and proximalization of the distal airspaces, as indicated by ectopic expression of the airway epithelial marker SOX2 (Figure 1B, boxed region, and Figure 1C). LQ/LQ mice demonstrated a 3.5-fold increase in lung injury compared with WT mice in response to bleomycin challenge (Figure 1D). LQ/LQ mice demonstrated an exacerbated fibrotic response with a 2.8-fold increase in collagen content measured by second harmonic imaging (Figure 1, B and E) and a 1.3-fold increase in hydroxyproline concentration (Figure 1F) compared with bleomycin-challenged WT mice. Consistent with increased lung collagen, bleomycin-challenged LQ/LQ mice developed restrictive lung disease, as indicated by a 35% decrease in lung compliance (Figure 1G) and a 1.5-fold increase in lung elastance (Supplemental Figure 3B); in contrast, lung function in bleomycin-challenged WT mice was similar to their unchallenged counterparts. Surfactant homeostasis was altered in bleomycin-challenged LQ/LQ mice with a 1.5-fold increase in both total phospholipids and saturated phosphatidylcholine levels in bronchoalveolar lavage fluid (BALF) compared with bleomycin-challenged WT mice (Supplemental Figure 3, C and D). Collectively, these data indicate that lung repair capacity following repeated injury is significantly compromised in LQ/LQ mice and is associated with the risk of persistent disease.

### Constitutive expression of the LQ allele is associated with impaired AT2 cell expansion in the postnatal lung.

**Figure 2:**
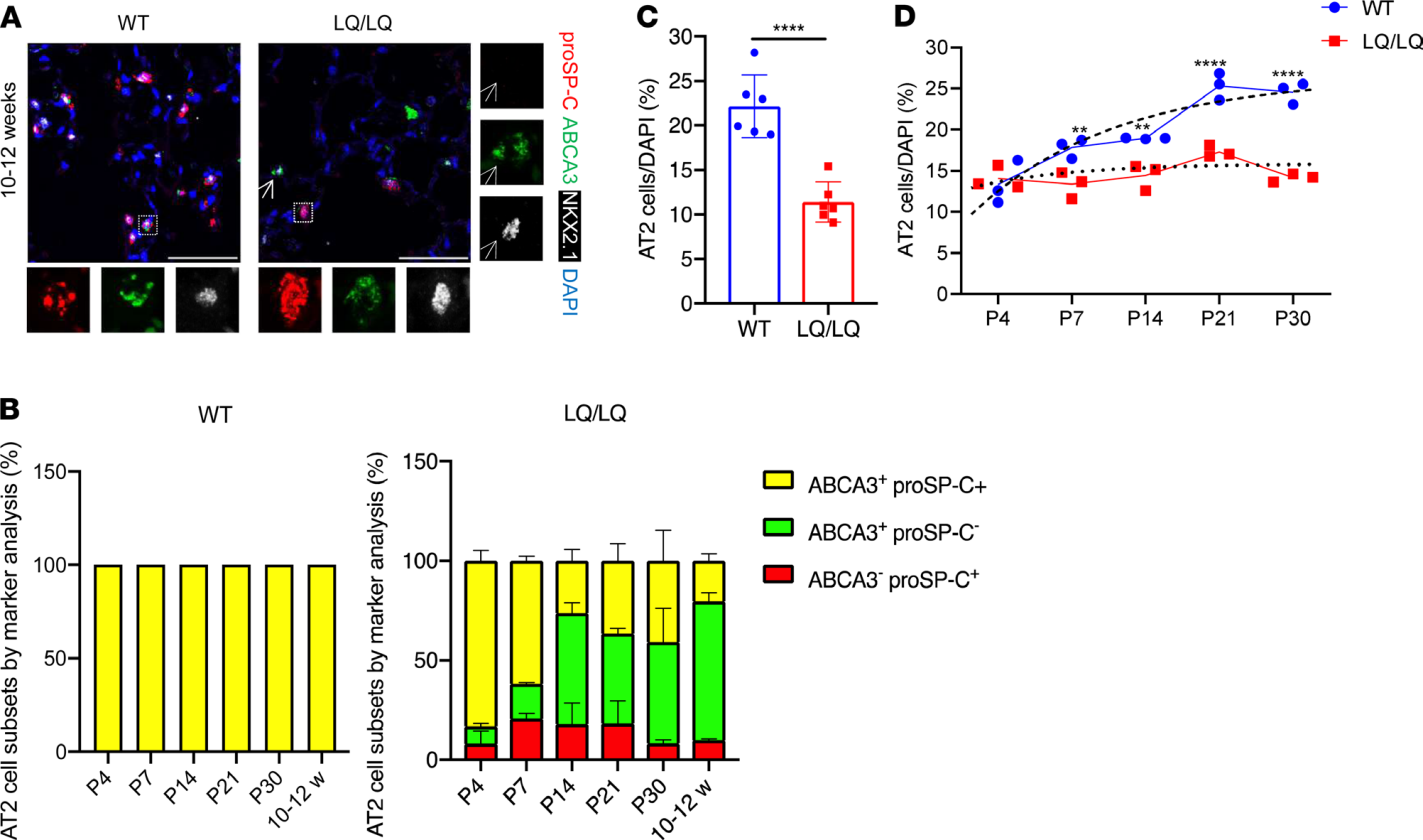
AT2 cell numbers are decreased in adult LQ/LQ mice. (**A**) Representative maximum intensity projections from confocal *Z*-stacks of lung sections obtained from 10- to 12-week-old mice stained with proSP-C, ABCA3, and distal epithelial cell transcription factor NKX2.1. Insets show individual channels for AT2 cell markers. Arrows point to NKX2.1^+^ABCA3^+^proSP-C^–^ AT2 cell. Scale bars: 50 μm. (**B**) Frequency distribution for AT2 cell markers, proSP-C, and ABCA3 in WT and LQ/LQ lung sections. All WT AT2 cells were proSP-C^+^ ABCA3^+^ at all time points. (**C**) Morphometric analyses of AT2 cells from 10- to 12-week-old mice. Each data point represents an individual mouse. ****P < 0.0001 by unpaired 2-tailed *t* test. (**D**) Morphometric analyses of AT2 cells in lung sections obtained during postnatal alveolarization. Dashed and dotted lines represent curve fits for WT (exponential) and LQ/LQ (linear) data points, respectively. **P < 0.01, ****P < 0.0001 by 1-way ANOVA with Tukey’s multiple comparison test. DAPI counts remained similar between genotypes (Supplemental Figure 4A).

To determine the cause of increased susceptibility of LQ/LQ mice to injury, lung sections from unchallenged adult LQ/LQ mice were stained with proSP-C, ABCA3, and NKX2.1 to identify AT2 cells, the site of SP-C expression; antibodies directed to both N- and C-terminal peptides of proSP-C were used to verify results in LQ/LQ mice. Confocal imaging demonstrated largely peripheral, punctate localization of proSP-C in WT AT2 cells (Figure 2A, box) compared with perinuclear proSP-C accumulation in LQ/LQ AT2 cells (Figure 2A, box), consistent with retention of proSP-C^LQ^ in the ER. In WT mice, 100% of AT2 cells were NKX2.1^+^proSP-C^+^ and ABCA3^+^ in adult lung and at all postnatal time points analyzed (Figure 2B). Importantly, in LQ/LQ mice, a minor subset of NKX2.1^+^ABCA3^+^ cells with undetectable levels of proSP-C, was observed at P4 (Figure 2B). This population of SP-C^–^ AT2 cells increased at P14 and comprised 55.3% ± 16.6% of total AT2 cells in adult lung; therefore, for morphometric analyses, AT2 cells were defined as NKX2.1^+^, proSP-C^+^, and/or ABCA3^+^. The frequency of AT2 cells in adult LQ/LQ mice decreased by 48.4% compared with age-matched WT controls (Figure 2C), indicating that impaired lung repair in adult LQ/LQ mice was associated with an antecedent reduction in AT2 cell numbers.

To determine the onset of reduced AT2 cell frequency, morphometric analyses were performed on lung sections obtained from WT and LQ/LQ mice during postnatal alveolarization between P4 and P30. Postnatal expansion of WT AT2 cells occurred between P4 and P21, with the frequency of AT2 cells increasing 1.3-fold between P4 and P7, followed by a further 1.3-fold increase between P14 and P21 (Figure 2D). By P30, the frequency of WT AT2 cells was similar to that in adult lung, with an overall increase of 1.9-fold between P4 and P30 (Figure 2D). In contrast, no increase in AT2 cell frequency was detected in LQ/LQ mice between P4 and P14 (Figure 2D). LQ/LQ AT2 cells increased by 1.2-fold between P14 and P21; however, the frequency of AT2 cells at P30 was not significantly different from that at P4, resulting in an overall 42% decrease compared with WT mice (Figure 2D). Notably, the number of nuclei was comparable between WT and LQ, between P4 and P14, and was significantly decreased in LQ/LQ lungs at P21 and P30, indicating that AT2 cell frequency was not skewed by normalization to frequency of nuclei (Supplemental Figure 4A). Overall, constitutive expression of the LQ allele was associated with failure of AT2 cell expansion during alveolarization.

Postnatal expansion of AT2 cells stems from active proliferation, supported by mesenchymal signaling, which commenced at P4 in WT lungs (Supplemental Figure 4, B and C). AT2 cell proliferation was maintained at P7, decreased by 65% at P14, and ceased at P21 (0%) in WT lungs. In contrast, the proliferative index of LQ/LQ AT2 cells was relatively low at P4 (decreased by 66% compared with WT) and peaked at P7 (Supplemental Figure 4C). Proliferation of LQ/LQ AT2 cells decreased modestly at P14 and was barely detectable at P21. Comparable proliferative indices for WT and LQ/LQ AT2 cells between P7 and P21 suggest that both continual cell loss and decreased proliferation at P4 contribute to fewer AT2 cells in LQ/LQ mice throughout postnatal alveolarization. Collectively, these data indicate that expression of the LQ allele impairs postnatal AT2 cell expansion, resulting in a significant and permanent reduction in AT2 cell numbers in adult mice; furthermore, expression of the mutant allele was associated with delayed onset of AT2 cell proliferation.

### Impaired postnatal AT2 expansion is associated with a transient neonatal increase in proSP-C^LQ^ protein accumulation.

**Figure 3:**
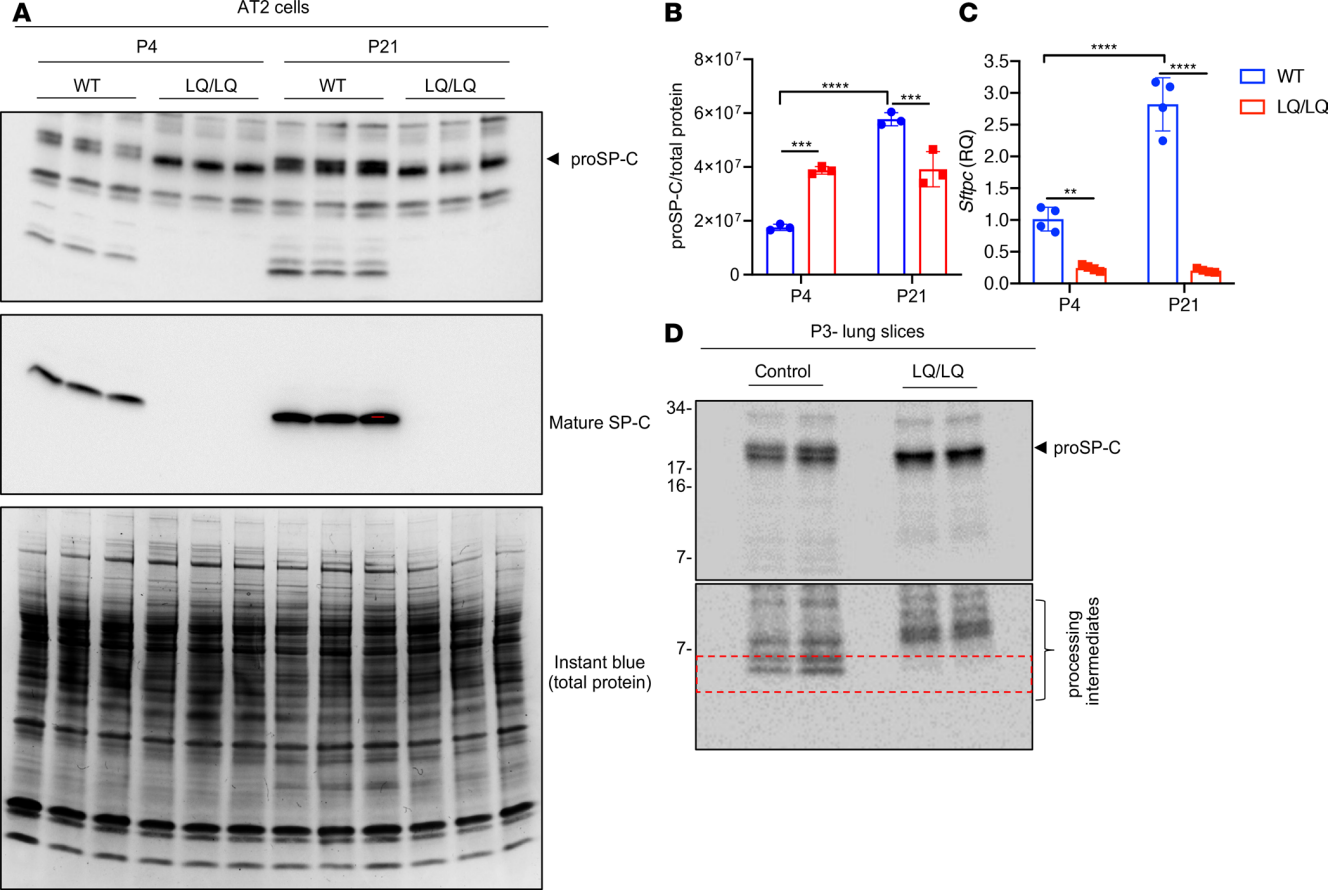
Accumulation of proSP-C in P4 LQ/LQ AT2 cells. (**A**) Western blot analyses of 20 μg of AT2 cell lysates separated by SDS-PAGE. Gel was stained after transfer with Coomassie-based instant blue to assess protein loading. (**B**) Normalization of proSP-C levels in **A** to total protein using Bio-Rad ImageLab software. (**C**) Relative *Sftpc* mRNA levels in isolated AT2 cells obtained by quantitative PCR of 20 ng of cDNA. Data were normalized to *Ppia* expression. Cycle threshold for *Ppia* was constant in all genotypes and across developmental time points (Supplemental Figure 5D). RQ, relative quantitation. For **B** and **C**, **P < 0.01, ***P < 0.001, ****P < 0.0001 by 2-way ANOVA with Sidak’s multiple comparison test. (**D**) Lung slices (1 mm) from P3 mice were labeled with [^35^S] methionine/cysteine for 30 minutes, followed by immunoprecipitation of 4.6 × 10^6^ counts with an antibody directed against the mature SP-C peptide (detects both SP-C proprotein and mature peptide). Lung homogenates were separated by SDS-PAGE, followed by phosphor imaging to detect newly synthesized proSP-C. Top and bottom panels were exposed for 2 and 7 days, respectively. Red box in the bottom portion shows absence of the mature peptide in LQ/LQ lung homogenates. Arrowheads in **A** and **D** point to SP-C proprotein. Unedited blots are available online with this article.

To determine if altered postnatal expansion of LQ/LQ AT2 cells was associated with changes in proSP-C^LQ^ expression, Western blot analyses were performed on equal numbers of AT2 cells isolated at time points bracketing the postnatal proliferative phase. At P4, proSP-C in LQ/LQ AT2 cells was increased 2.2-fold compared with WT proSP-C (Figure 3, A and B); SP-C^LQ^ was detected predominantly as a single proprotein band, consistent with trapping in the ER and failure of post-ER modifications, including proteolytic processing. The increase in proSP-C^LQ^ protein was observed as early as P1, where LQ/LQ AT2 cells demonstrated a 2.9-fold increase in proSP-C levels compared with WT cells (Supplemental Figure 5, A and B). At P21, proSP-C^LQ^ protein levels in LQ/LQ AT2 cells were unchanged compared with P4; in contrast, proSP-C in WT AT2 cells increased 3.3-fold between P4 and P21, resulting in significantly higher levels compared with LQ/LQ AT2 cells (Figure 3, A and B). Processing of proSP-C to its mature peptide was detected at all time points in WT but was inhibited in LQ/LQ AT2 cells (Figure 3A and Supplemental Figure 5A). Importantly, in the absence of a challenge, neither the lack of mature peptide or the loss of AT2 cells was associated with changes in lung function or surfactant homeostasis in LQ/LQ lungs (control data in Figure 1G and Supplemental Figure 3, B–D).

Unexpectedly, the neonatal increase in proSP-C^LQ^ protein occurred independently of mRNA levels. *Sftpc* expression in LQ/LQ AT2 cells was only 24% of WT levels at P4 (Figure 3C). At P21, there was a 2.8-fold increase in WT *Sftpc* expression that corresponded with the temporal increase in WT proSP-C protein levels (Figure 3C); however, a similar increase in *Sftpc* expression was not observed in LQ/LQ AT2 cells (Figure 3C). These data indicate a discordance between abundance and subsequent translation of the *Sftpc*^LQ^ mRNA.

In the absence of elevated *Sftpc* mRNA levels, increased proSP-C^LQ^ protein could arise from altered translation and/or degradation of SP-C in the P1–P4 developmental window. To assess synthesis of the proSP-C, lung slices from P3 mice were metabolically labeled with [^35^S] methionine/cysteine, followed by immunoprecipitation with an antibody that detects both the proprotein and mature peptide. Even though *Sftpc* expression was significantly lower in LQ AT2 cells, levels of newly synthesized proSP-C were higher in LQ/LQ lung slices compared with WT (Figure 3D). The mature peptide was absent from radiolabeled LQ/LQ samples, consistent with the degradation of misfolded proSP-C^LQ^ and consequent inhibition of mature peptide generation. To determine if increased synthesis of proSP-C^LQ^ at P4 occurred due to an increase in overall protein synthesis, isolated AT2 cells were labeled with the amino acid analog L-azidohomoalanine, followed by ligation with biotin alkyne to detect newly synthesized proteins. Western blot analyses of biotinylated proteins indicated that levels of nascent proteins in LQ/LQ AT2 cells were comparable with WT AT2 cells at both P4 and P21 (Supplemental Figure 6). These results indicate that global protein synthesis in P4 LQ/LQ AT2 cells cannot fully account for increased proSP-C^LQ^ synthesis, suggesting additional, and as yet unidentified, posttranscriptional regulation of *Sftpc*^LQ^; the unique, transient neonatal increase in proSP-C^LQ^ protein is associated with increased translation of the LQ allele that occurs prior to impaired postnatal AT2 cell expansion.

### Increased neonatal accumulation of proSP-C^LQ^ is associated with activation of oxidative stress.

**Figure 4:**
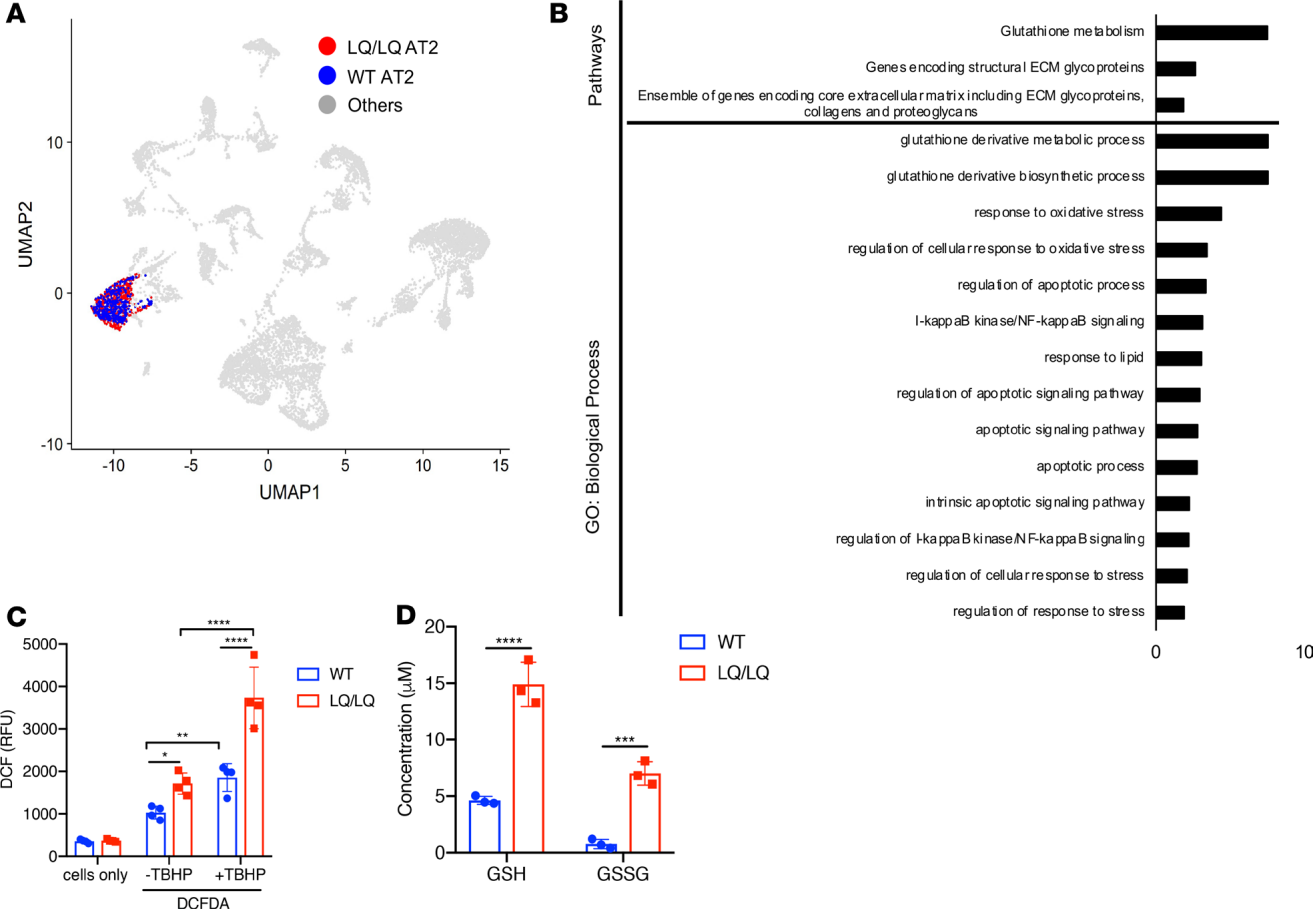
Activation of oxidative stress in P4 LQ/LQ AT2 cells. (**A**) UMAP embedding shows AT2 cell clusters (*n*, WT = 415 and LQ/LQ = 540) obtained from scRNAseq of P4 lungs. (**B**) Toppfun analyses show pathways and biological processes characterizing the P4 LQ/LQ AT2 cell population. Groups are ordered by descending *P* values (*x* axis, –log_10_ of *P* value). (**C**) Freshly isolated P4 AT2 cells were incubated with DCFDA for measurement of ROS levels. Cells without DCFDA were used as a control to measure background fluorescence. RFU, relative fluorescence unit; TBHP, tert-butyl hydrogen peroxide (inducer of oxidative stress). (**D**) Glutathione (GSH) concentrations were measured in freshly isolated, deproteinized P4 AT2 cells. AT2 cell lysates were incubated with 2-vinylpyridine for measurement of oxidized glutathione disulfide (GSSG). For **C** and **D**, *P < 0.05, **P < 0.01, ***P < 0.001, ****P < 0.0001 by 2-way ANOVA with Sidak’s multiple comparison test.

To identify differences in gene expression and cell type composition associated with increased proSP-C^LQ^ translation, single cell RNA sequencing (scRNAseq) was performed on P4 lungs using the 10× genomics platform. A total of 12,652 single cells was analyzed from P4 lungs (Supplemental Figure 7A), and unsupervised clustering revealed 52 cell clusters and 15 major cell types (Supplemental Figure 7B). In total, 415 and 540 AT2 cells were identified from WT and LQ/LQ P4 lungs, respectively (Figure 4A). AT2 cell–specific differentially expressed genes were identified using a binomial based differential expression test. Genes expressed higher in WT (*n* = 1032) were functionally associated with lipid metabolism, principally cholesterol biosynthesis and SREBP signaling (Supplemental Figure 7C). In contrast, genes induced in LQ/LQ AT2 cells (*n* = 191) were functionally associated with oxidative stress (particularly glutathione metabolism), NF-κB signaling, and apoptosis (Figure 4B). To determine if the robust oxidative stress signature was associated with increased levels of reactive oxygen species (ROS), isolated AT2 cells were incubated with 2′,7′-dichlorofluorescin diacetate (DCFDA). DCFDA is deactelyated by cellular esterases and subsequently oxidized to the highly fluorogenic 2′,7′-dichlorofluorescein (DCF) by ROS; fluorescence emitted by DCF is directly proportional to the amount of cellular ROS. P4 LQ/LQ AT2 cells demonstrated a 1.7-fold increase in ROS levels compared with WT AT2 cells at baseline (Figure 4C). Furthermore, LQ/LQ AT2 cells were exquisitely sensitive to addition of tert-Butyl hydroperoxide (TBHP), an inducer of oxidative stress, resulting in a 2-fold increase in ROS levels compared with TBHP-treated WT AT2 cells (Figure 4C). Sensitivity to TBHP treatment was maintained in P21 LQ/LQ AT2 cells, with a 2-fold increase in ROS levels compared with WT AT2 cells (Supplemental Figure 7D); however, no significant differences in ROS levels were observed at baseline for P21 WT and LQ/LQ AT2 cells. Consistent with increased oxidative stress, P4 LQ/LQ AT2 cells demonstrated a 3.2- and 9.1-fold increase in glutathione (GSH) and glutathione disulfide (GSSG) levels, respectively (Figure 4D), decreasing the GSH/GSSG ratio by 66% in LQ/LQ AT2 cells compared with WT. The increase in GSH and GSSG levels was maintained in LQ/LQ AT2 cells at P21 with a 4-fold and 2.8-fold increase in GSH and GSSG levels, respectively, compared with WT (Supplemental Figure 7E); however, the GSH/GSSG ratio in LQ/LQ AT2 cells was comparable with that of WT at P21, consistent with resolution of oxidative stress. Overall, increased neonatal translation of proSP-C^LQ^ protein was associated with a strong oxidative stress response that transiently altered redox balance in postnatal AT2 cells.

### Oxidative stress impacts AT2 cell survival and differentiation.

**Figure 5:**
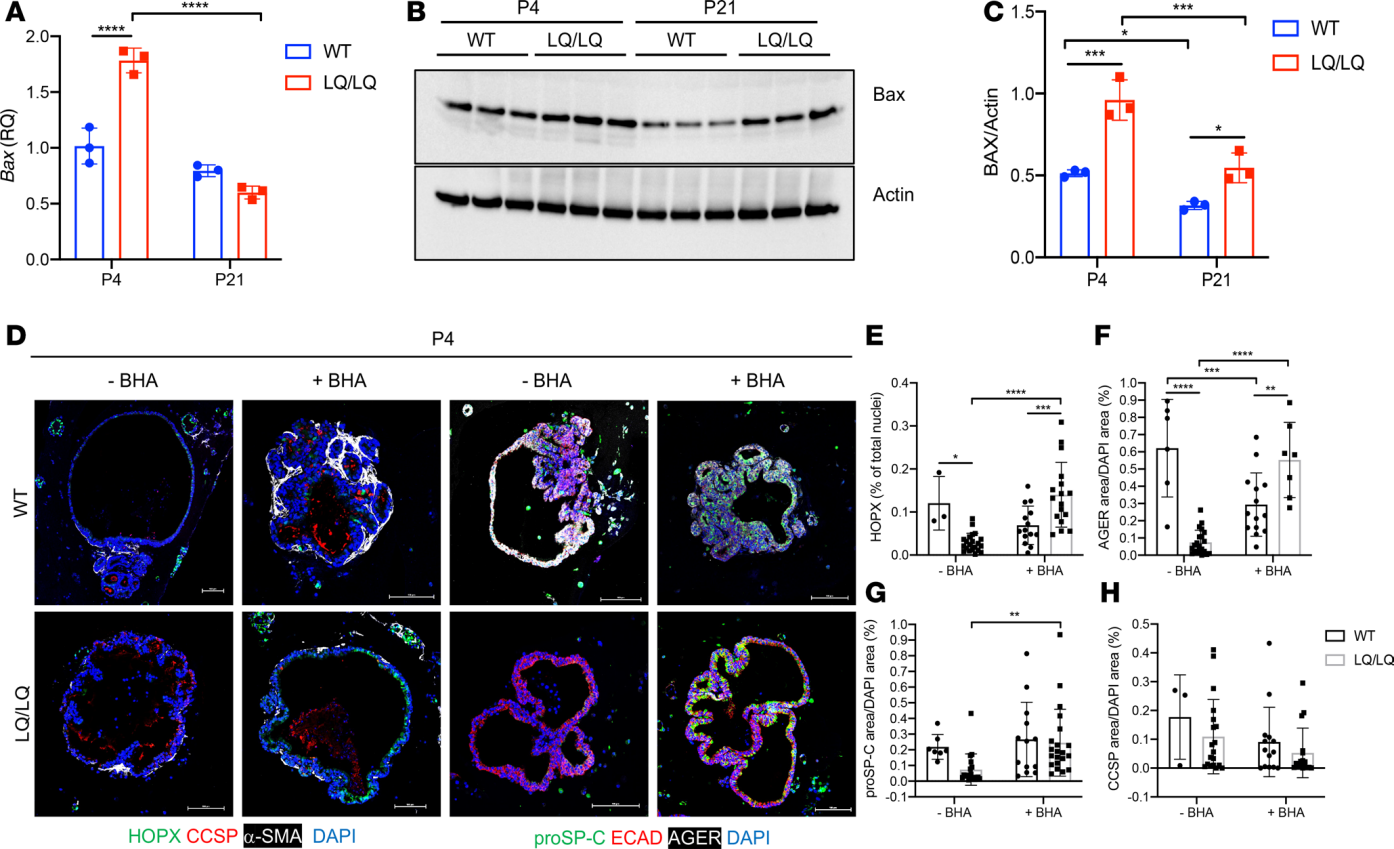
Oxidative stress is associated with proapoptotic signaling and impaired differentiation. (**A**) Relative *Bax* mRNA levels in isolated AT2 cells obtained by quantitative PCR of 25 ng of cDNA. Data were normalized to *Ppia* expression. (**B**) Western blotting for proapoptotic protein, BAX. In total, 30 μg of AT2 cell lysates was separated by SDS-PAGE. (**C**) Densitometry for BAX levels in **B**. Data were normalized to Actin. (**D**) Confocal images of organoids generated from P4 WT and LQ/LQ AT2 cells. Organoid cultures were treated with or without the antioxidant, butylated hydroxyanisole (BHA). HOPX, AT1 cell marker; CCSP, club cell (proximal airway epithelial cell) marker; α-SMA: α-smooth muscle actin; proSP-C, AT2 cell marker; ECAD, pan-epithelial cell marker; AGER, AT1 cell marker. Scale bars: 100 μm. (**E**–**H**) Morphometric analyses of HOPX^+^ (**E**), AGER^+^ (**F**), proSP-C^+^ (**G**), and CCSP^+^ (**H**) organoids (circles, WT; squares, LQ/LQ). For **A**, **C**, and **E**–**H**, *P < 0.05, **P < 0.01, ***P < 0.001, ****P < 0.0001 by 2-way ANOVA with Sidak’s multiple comparison test. Unedited blots are available online with this article.

Augmented production of ROS and imbalanced redox states are associated with activation of apoptosis. Single cell explorer (20), a recently developed algorithm for scRNAseq analysis and visualization of enriched bioprocess and signaling pathways on t-stochastic neighbor embedding (t-SNE) or uniform manifold approximation and projection (UMAP) plots, identified a strong stress and cell death signature in the P4 LQ/LQ AT2 cell cluster, including the ER-associated degradation (ERAD), UPR, proteasome, and apoptosis pathways compared with the WT AT2 cell cluster (Supplemental Figure 8). Quantitative PCR (qPCR) demonstrated a 1.8-fold increase in the expression of the proapoptotic gene *Bax* in P4 LQ/LQ AT2 cells that resolved to WT levels by P21 (Figure 5A). Western blotting of isolated AT2 cells demonstrated a 1.9-fold increase in BAX protein levels in P4 LQ/LQ AT2 cells compared with WT (Figure 5, B and C). Although BAX protein decreased by 45% in P21 LQ/LQ AT2 cells compared with P4, levels remained elevated by 1.3-fold compared with P21 WT, suggesting that apoptotic signaling may be sustained.

To determine if oxidative stress negatively impacted AT2 cell differentiation, ex vivo organoids were generated from AT2 cells isolated from P4 lungs cocultured with adult mouse lung fibroblasts (Figure 5D). Consistent with previous studies (21), P4 WT AT2 cells differentiated into AT1 cells in organoid cultures; however, organoids generated from P4 LQ/LQ AT2 cells demonstrated relatively little expression of AT1 markers HOPX and AGER (Figure 5D). Importantly, treatment of LQ/LQ organoids with the antioxidant butylated hydroxyanisole (BHA) resulted in a 7.4-fold and 4.8-fold increase in AGER^+^ and HOPX^+^ cells, respectively (Figure 5, E and F). Expression of proSP-C increased by 3.4-fold in BHA-treated LQ/LQ organoids (Figure 5G). Notably, the number and size of organoids generated from WT and LQ/LQ AT2 cells remained unchanged with or without BHA treatment (Supplemental Figure 9, A–C). Collectively, these results suggest that oxidative stress arising from a transient neonatal increase in proSP-C^LQ^ protein levels inhibits postnatal survival, expansion, and differentiation of AT2 cells. Importantly, expression of proximal epithelial marker CCSP remained unchanged with BHA treatment (Figure 5H), indicating that antioxidant treatment of LQ/LQ organoids selectively restored key aspects of distal alveolar epithelial cell maturation.

### AT2 cell stress is associated with impaired macrophage development and M2 polarization.

To determine if activation of apoptotic signaling in AT2 cells at P4 impacted development and differentiation of other cell types during postnatal alveolarization, scRNAseq was performed on P21 lungs using the 10× genomics platform. A total of 15,218 cells and 14 major cell clusters were identified, which included 1229 WT and 443 LQ/LQ AT2 cells. scRNAseq data sets obtained from P4 and P21 lungs were integrated to analyze potential temporal changes in cell-type composition and gene expressions (Supplemental Figure 10A); analyses were focused to immune cells — particularly macrophages (Supplemental Figure 10, B–D), since the development and maturation of alveolar macrophages occurs coincident to AT2 cell expansion during postnatal alveolarization (22, 23). Gene expression analyses of markers associated with the alveolar and interstitial macrophage subsets demonstrated reduced expression in P4 LQ/LQ lungs compared with WT; expression of marker genes resolved to WT levels in P21 LQ/LQ lungs (Supplemental Figure 10E). Restoration of marker expression was observed as early as P14, where flow cytometric analyses demonstrated comparable numbers of alveolar macrophages (Supplemental Figure 11A). Consistent with scRNAseq analyses, comparable expression of CD163 and CD206 (encoded by MRC1) was identified in alveolar macrophages from P14 LQ/LQ mice, compared with WT mice. Finally, the frequency of Arginase1^+^ alveolar macrophages was increased 5-fold and 7.1-fold in the lungs and BALF of P14 LQ/LQ mice, respectively, compared with WT mice (Supplemental Figure 11, B and C), suggesting alternative activation of macrophages and induction of an anti-inflammatory response. Collectively, these data suggest that oxidative stress in neonatal AT2 cells impacts crosstalk with the immune cells, resulting in suppression of macrophage development; resolution of development occurs concurrent with M2 polarization of alveolar macrophages.

### Chronic stress promotes adaptation in P21 LQ/LQ AT2 cells.

**Figure 6:**
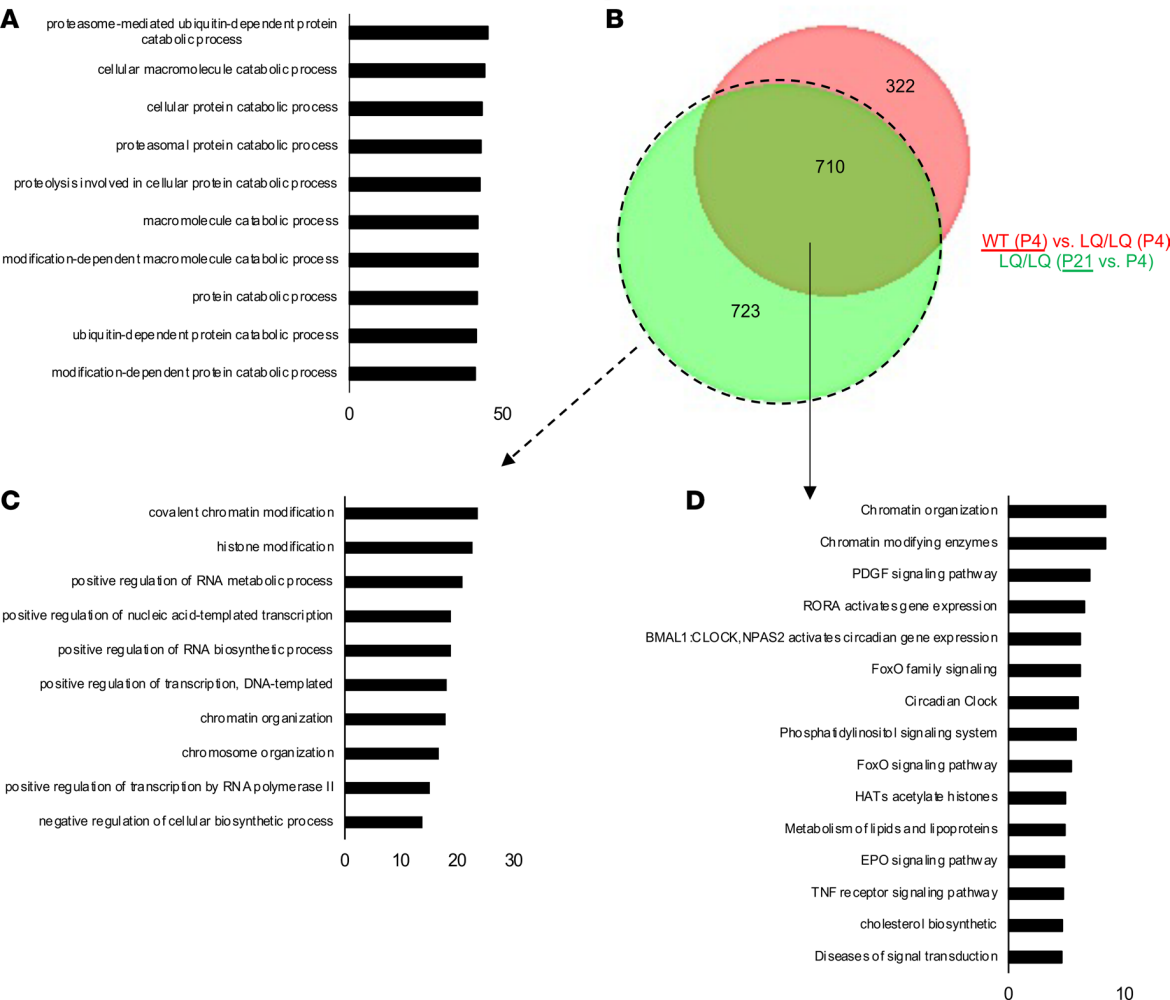
Chronic stress promotes adaptation in P21 LQ/LQ AT2 cells. (**A**) GO analyses of proteomics data show overrepresented processes in P4 LQ/LQ AT2 cells compared with P4 WT AT2 cells. Biological processes are ordered by descending *P* values (*x* axis, –log_10_ *P* value). (**B**) Venn diagram representing the overlap between genes characterizing the P21 LQ/LQ and P4 WT AT2 cell populations compared with P4 LQ/LQ AT2 cell population. (**C**) Toppfun analyses show pathways characterizing the P21 LQ/LQ AT2 cells compared with P4 LQ/LQ AT2 cells. (**D**) Toppfun analyses show pathways characterizing the common genes (710 genes) from **B**. For **C** and **D**, pathways are ordered by descending *P* values (*x* axis, –log_10_ *P* value).

We hypothesized that activation of stress responses at P4 promoted adaptation, thereby allowing for survival of LQ/LQ AT2 cells at and after P21. Consistent with the scRNAseq data set, proteomic analyses of isolated P4 AT2 cells confirmed activation of a robust stress signature marked by an abundance of proteins associated with catabolism and, in particular, the proteasome pathway (*n* = 726 proteins) in P4 LQ/LQ AT2 cells (Figure 6A and Supplemental Figure 12A); in contrast, proteins associated with metabolic processes (*n* = 391 proteins) were more abundant in P4 WT AT2 cells (Supplemental Figure 12B). Consistent with an adaptive response, analyses of the scRNAseq data set at P21 revealed minimal differences between the WT and LQ/LQ AT2 population, indicating that the P21 LQ/LQ AT2 cells shared a similar transcriptome to that of WT AT2 cells. Furthermore, transcriptomic similarity was observed between P21 LQ/LQ AT2 cells and P4 WT AT2 cells (Figure 6B), suggesting delayed maturation of LQ/LQ AT2 cells. P21 LQ/LQ AT2 cell population was characterized by genes (*n* = 723) primarily associated with chromatin and RNA modification (Figure 6C); in addition, P21 LQ/LQ and P4 WT AT2 cells were commonly characterized by genes (*n* = 710) associated with chromatin modification, PI3 kinase, PDGF, and FOXO signaling pathways and lipid biosynthesis compared with P4 LQ/LQ AT2 cells (Figure 6D).

**Figure 7:**
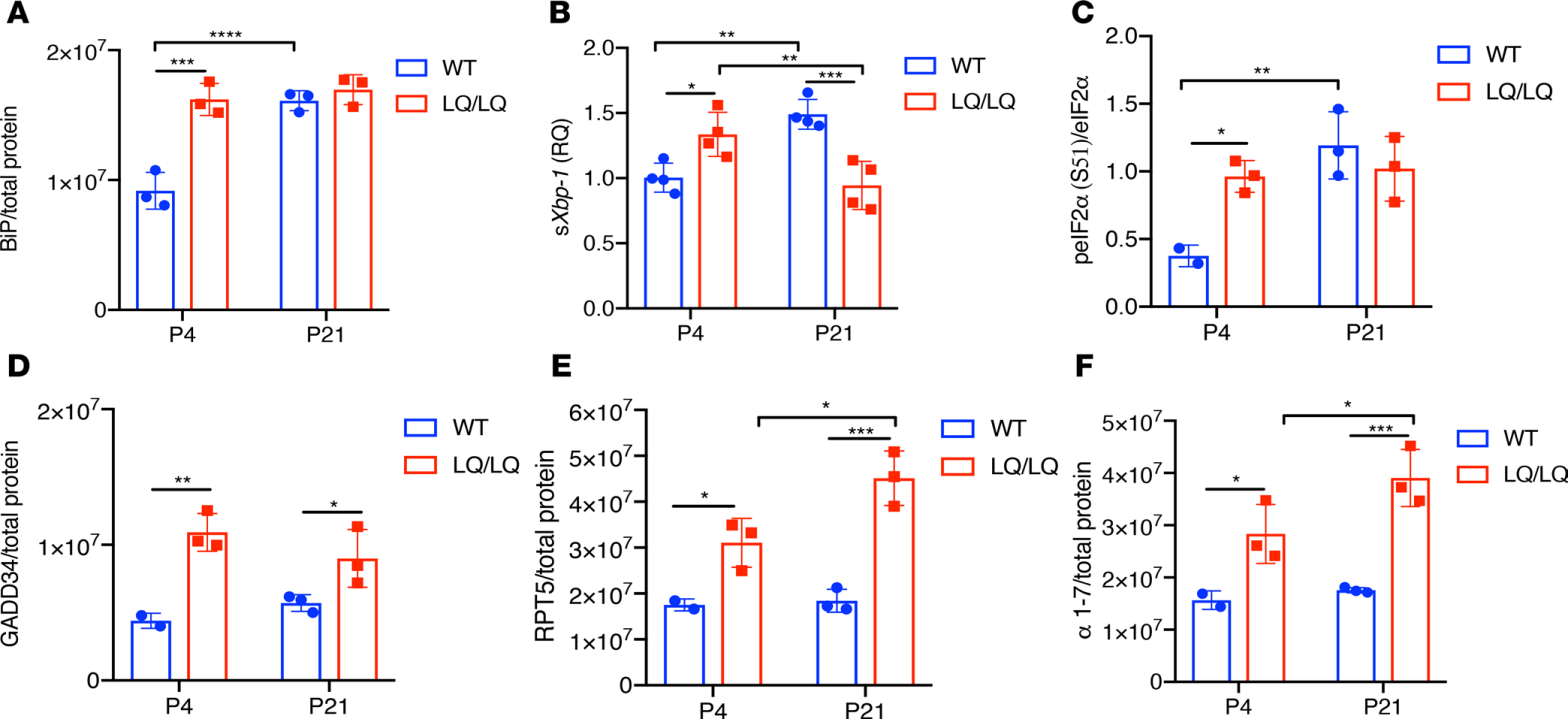
Resolution of stress responses in P21 LQ/LQ AT2 cells. (**A**) BiP protein levels normalized to total protein in isolated AT2 cells (blot shown in Supplemental Figure 13A). (**B**) Relative expression of spliced Xbp1 (*sXbp1*) mRNA obtained by quantitative PCR of 20 ng of AT2 cell cDNA. Data were normalized to *Ppia* expression (shown in Supplemental Figure 5D). (**C**) Ratio of peIF2α to total eIF2α levels in isolated AT2 cells (blot shown in Supplemental Figure 13B). (**D**) GADD34 protein levels normalized to total protein in isolated AT2 cells (blot shown in Supplemental Figure 13C). (**E** and **F**) Levels of proteasome subunits RPT5 (**E**) and α 1-7 (**F**) normalized to total protein in isolated AT2 cells (blot shown in Supplemental Figure 13D). For **E** and **F**, *P < 0.05, **P < 0.01, ***P < 0.001, ****P < 0.0001 by 2-way ANOVA with Sidak’s multiple comparison test.

To confirm resolution of stress responses and adaptation in LQ/LQ AT2 cells, Western blot analyses of select stress markers were performed in AT2 cells isolated at P4 and P21. Given the strong ERAD signature observed by scRNAseq of P4 LQ/LQ AT2 cells (Supplemental Figure 8), markers associated with the UPR, and proteasome pathways were chosen for validation. Western blot analyses of the master ER chaperone BiP indicated a 1.8-fold increase in P4 LQ/LQ AT2 cells compared with WT (Figure 7A and Supplemental Figure 13A), confirming activation of ER stress. Importantly, BiP levels were similar in P1 WT and LQ/LQ AT2 cells (Supplemental Figure 5, A and C), indicating that further activation of UPR occurred after this time point. A significant increase in spliced *Xbp1* (*sXbp1*) expression in P4 LQ/LQ AT2 cells compared with WT cells confirmed activation of the IRE1α branch of the UPR (Figure 7B). Western blot analyses also demonstrated a 2.7- and 2.3-fold increase in phosphorylated eIF2α and GADD34 levels, respectively, in LQ/LQ AT2 cells compared with WT AT2 cells at P4, consistent with activation of the PERK branch of the UPR and the integrated stress response (ISR) (Figure 7, C and D, and Supplemental Figure 13, B and C). LQ/LQ AT2 cells demonstrated augmented expression of proteasome subunits (Figure 7, E and F, and Supplemental Figure 13D), validating activation of the ERAD pathway and proteasome-mediated clearance of the misfolded proSP-C at P4.

By P21 there were significantly fewer AT2 cells in LQ/LQ lungs (Figure 2D); furthermore, more than half of surviving LQ/LQ AT2 expressed little or no detectable SP-C, whereas all WT AT2 robustly expressed SP-C (Figure 2B). These results suggest that adaptation involves loss of SP-C–expressing AT2 cells, as well as diminished translation and/or increased degradation of mutant proprotein. Consistent with enhanced clearance of misfolded SP-C, there was a significant increase in the expression of proteasome subunits RPT5 and α 1–7, compared with both P4 LQ/LQ and P21 WT AT2 cells (Figure 7, E and F, and Supplemental Figure 13D). Importantly, GADD34 levels remained elevated at P21 (Figure 7D and Supplemental Figure 13C), consistent with sustained PERK activation and delayed recovery of general translation in cells that survive chronic ER stress (24). BiP and peIF2α levels remained elevated at P21 but were now similar in WT and LQ/LQ AT2 (Figure 7, A and C, and Supplemental Figure 13, A and B), likely related to increased surfactant lipid synthesis associated with AT2 cell maturation. Interestingly, s*Xbp1* expression decreased by 30% in P21 LQ/LQ AT2 cells compared with P4 LQ/LQ AT2 cells (Figure 7B), consistent with a switch to an adaptive response where IRE1 signaling was attenuated during persistent ER stress (25). Collectively, these results suggest that both increased degradation and decreased translation may contribute to diminished proSP-C load in LQ/LQ AT2 cells at P21. The varied temporal course of activation of the PERK and IRE1α branches of the UPR influences the fate of AT2 cells in the face of chronic ER stress; a subpopulation of LQ/LQ AT2 cells emerge at P21 that survive and adapt to the stress imposed by expression of mutant proSP-C^LQ^.

## Discussion

Constitutive expression of a disease-associated allele encoding a misfolded form of proSP-C (L184Q) resulted in activation of oxidative stress that was linked to a transient increase in *Sftpc* mRNA translation in neonatal mice. Subsequent induction of multiple cell stress pathways was associated with impaired expansion of AT2 cells during postnatal alveolarization, resulting in a permanent loss of AT2 cells and, ultimately, decreased regenerative capacity in the adult lung.

Expression of allele variants encoding SP-C have been linked to cytotoxicity and pathogenesis in vivo. Prenatal expression of a Δexon4 *SFTPC* transgene (9) or the C121G *Sftpc* allele (17) resulted in AT2 cell death, disrupted lung morphogenesis, and neonatal lethality. These mouse models confirmed in vitro findings of mutant SP-C toxicity but failed to model the human disease, which is not typically associated with postnatal lethality. Acute induction of expression of the I73T (26) or C121G *Sftpc* allele in adult lung resulted in interstitial lung disease marked by alveolitis, aberrant remodeling, and restrictive lung disease. These mouse models strongly linked expression of the mutant *Sftpc* allele to development of fibrotic lung disease, and they model the acute exacerbation phase of disease. Furthermore, acute induction of an L188Q *SFTPC* transgene in adult lung was associated with decreased repair capacity following lung injury. In the current study, we confirm that constitutive (as opposed to induced) expression of the L184Q *Sftpc* allele is also associated with impaired lung regeneration, and we link the adult phenotype to permanent loss of AT2 cells during postnatal alveolarization. The current mouse model suggests that adult susceptibility to injury is established in the postnatal period and provides a potential explanation for the delayed onset of disease in patients with familial pulmonary fibrosis.

*Sftpc* mRNA in LQ/LQ mice was expressed at one-fourth of WT levels in both postnatal and adult AT2 cells. Similarly, in all mouse models to date, mutant *Sftpc* alleles were consistently expressed at lower levels than WT *Sftpc*, despite significant differences in targeting strategies and construct design (15, 17, 26). These results suggest that mutations in this locus might suppress *Sftpc* expression, although the mechanism underlying putative allele suppression and whether allele suppression occurs in human patients with *SFTPC* mutations are unknown. Importantly, there was significant discordance between *Sftpc* mRNA and protein levels in postnatal LQ/LQ AT2 cells, such that misfolded proSP-C^LQ^ accumulated to significantly higher levels than in WT AT2 cells; this observation is consistent with increasing findings that mRNA abundance is a poor predictor of protein abundance and that translational control is a critical step in the regulation of posttranscriptional gene expression (27).

Consistent with perinatal maturation of the surfactant system, AT2 and AT1/AT2 bipotent cells experience transient lipid stress and UPR activation at P1 (28); cell stress in this critical developmental window is further augmented by expression of the LQ allele. Increased neonatal synthesis of mutant proSP-C resulted in activation of multiple stress pathways, including ER stress, UPR, oxidative stress, and the ISR, coincident with commencement of postnatal alveolarization at P4. Resolution of stress responses toward the culmination of alveolarization was accompanied by decreased frequency of surviving AT2 cells, which, in turn, was associated with impaired lung repair in adult LQ/LQ mice. Overall, these data are consistent with the hypothesis that stress arising from postnatal expression of the LQ allele, superimposed on stress associated with normal AT2 cell maturation, leads to a transient increase in proSP-C^LQ^ synthesis and permanent loss of AT2 cells. Whether these cell-stress events are unique to the developing lung or are recapitulated during lung regeneration remains an important and unanswered question.

Increased neonatal synthesis of proSP-C^LQ^ was associated with generation of ROS and altered glutathione levels. Previous studies have demonstrated that defective folding of proteins in the ER lumen resulted in activation of the UPR and oxidative stress, leading to apoptosis (29, 30). Dysregulated translation in mice with disrupted eIF2α phosphorylation resulted in increased ROS generation and β cell failure (31). Furthermore, increased protein synthesis downstream of the eIF2α pathway was identified as a critical step required for recovery of cells from acute stress (32, 33); however, increased protein synthesis without restoration of homeostasis resulted in generation of ROS. In the present study, global protein synthesis was comparable between LQ/LQ and WT AT2 cells, and increased translation of proSP-C^LQ^ was associated with oxidative stress and apoptosis. Given that proSP-C is an extraordinarily hydrophobic and highly abundant secretory protein, increased translation of the misfolded proprotein likely rapidly overwhelms the ER protein folding and ERAD machinery in AT2 cells. Importantly, all AT2 cells in LQ/LQ mice expressed high levels of proSP-C at P4; in contrast, there was a significant increase in the frequency of proSP-C^–^ABCA3^+^ AT2 cells at P21 and beyond, with resolution of oxidative stress and normalization of the GSH/GSSG ratio at P21 in LQ/LQ AT2 cells. These results identify the temporal emergence of distinct AT2 cell populations in which a subpopulation suppresses proSP-C^LQ^ expression (proSP-C^lo^) and a separate subpopulation maintains high levels of proSP-C^LQ^ (proSP-C^hi^). We propose that proSP-C^hi^ AT2 cells fail to adapt to chronic stress resulting from constitutive *Sftpc^LQ^* expression and are eventually eliminated by apoptosis.

Surviving proSP-C^lo^ AT2 cells were characterized by genes associated with lipid biosynthetic pathways, suggesting that adaptation to elevated postnatal cell stress promoted AT2 cell maturation and suppression of proSP-C^LQ^ expression. Similar adaptation to chronic ER stress involving an increase in lipid synthesis and metabolism was recently reported in thyrocytes expressing misfolded thyroglobulin (34). Oxidative stress and ER stress are known to promote formation of stress granules and P-bodies mediated in part by phosphorylation of eIF2α (35); stress granules sequester untranslated mRNA and are involved in mRNA storage and degradation (36). A recent study demonstrated the coordinated involvement of VCP/p97 and the proteasome in ribosome release and partitioning of mRNA into stress granules under conditions of stress (37); whether similar stress granules form and sequester *Sftpc*^LQ^ in P21 LQ/LQ AT2 cells remains an important unanswered question.

Unresolved ER and oxidative stress disrupt cellular physiology and survival (38). Notably, ER stress associated with insulin mutations resulted in reduced β cell mass with no apparent apoptosis of β cells (39, 40); exposure to ER stress during the postnatal period considerably reduced expansion, differentiation, and functional maturation of β cells in the *Akita* mouse model (40). LQ/LQ AT2 cells demonstrated a strong apoptosis signature by scRNAseq analyses and decreased proliferation at P4. Although proliferation normalized in LQ/LQ AT2 cells by P7, reduced frequency of AT2 cells between P7 and P21 indicates that proliferation was insufficient to compensate for AT2 cell loss. Expression of a proapoptotic marker was elevated in LQ/LQ AT2 cells; however, we were unable to identify apoptotic AT2 cells in LQ/LQ lungs, likely due to rapid clearance of dead cells by macrophages.

Limited AT2 to AT1 cell differentiation was observed in organoids derived from P4 LQ/LQ lungs, consistent with the scRNAseq data that demonstrated that processes associated with AT2 cell maturation and development were underrepresented in P4 LQ/LQ AT2 cells compared with WT. Antioxidant treatment of LQ/LQ organoids restored significant AT1 differentiation and AT2 maturation, suggesting that oxidative stress impairs alveolar epithelial differentiation. The precise mechanisms underlying defective differentiation in P4 LQ/LQ organoids and whether differentiation is similarly impaired in adult LQ/LQ AT2 cells merit further investigation.

L184Q mice provide a physiological model of chronic stress response with temporally distinct molecular and cellular phenotypes directly proportional to misfolded proSP-C^LQ^ levels and activation of oxidative stress. Interestingly, neonatal mice challenged with hyperoxia between P0 and P4 demonstrated a 50% reduction in *Sftpc*^+^ AT2 cells in adulthood with minimal effect on alveolar homeostasis, similar to L184Q mice (41–43). Furthermore, lung regeneration was significantly impaired in response to bleomycin challenge (42) or influenza A virus (IAV) infection (44), resulting in enhanced recruitment of airway progenitors. Collectively, these results support the hypothesis that neonatal exposure to intrinsic or extrinsic oxidative stress impairs postnatal AT2 cell expansion, leading to permanent loss of AT2 cells and dysfunctional lung repair in adult mice. These findings raise the intriguing possibility that postnatal antioxidant therapy may restore AT2 endowment and enhance lung repair in patients carrying BRICHOS mutations.

## Methods

Supplemental Methods are available online with this article.

### Mice.

The 551 T→A point mutation (L184Q substitution) was generated by site-directed mutagenesis (QuikChange, Stratagene) of the mouse SP-C (*Sftpc*) cDNA. Three stop codons, bovine growth hormone polyadenylation signal (BGH-pA), and a 3′ AscI site were subsequently incorporated into the cassette encoding the cDNA. To generate the 5′ arm, 3314 bp of the *Sftpc* promoter were amplified from WT ES cell genomic DNA (Taffy SvEvTAC 129 substrain), cloned downstream of a 5′ AscI site and subsequently built onto the mutated cDNA. To generate the 3′ arm, a *Sftpc* sequence from the beginning of intron 1 to exon 6+3361 nucleotides flanked by NotI sites was cloned from WT ES cell genomic DNA. The 3′ and 5′ arms were subcloned into the NotI and AscI sites of the OSdupdel2 vector (a gift from Oliver Smithies, University of North Carolina, Chapel Hill, North Carolina, USA). MLE12 cells were transfected with the complete construct, and *Sftpc* mRNA levels were examined by PCR electrophoresis. Linearized plasmid DNA (AclI site) was submitted to the University of Cincinnati ES Cell Core for electroporation. Clones were selected for resistance to neomycin, and recombination was verified by loss of PGK-TK cassette. Positive ES cell clones were injected into C57BL/6 blastocysts and implanted into pseudopregnant female mice. The resulting chimeras were mated to C57BL/6 mice to generate founder mice, which were screened for the presence of a single (heterozygous) knock-in allele. Subsequently, the neomycin resistance cassette (neo) was removed in vivo by crossing founder mice to the *EIIa*-Cre mice (the Jackson Laboratory). Excision of the cassette was confirmed by PCR using primers P1 (exon 1), 5′-TGG ACA TGA GTA GCA AAG AGG TC-3′; P3 (neomycin cassette), 5′-AGT TCT TCT GAG GGG ATC AAT TC-3′; and P4 (intron 1), 5′-ATC CTA AAA GCC CAA TCC TAA GC-3′. F1 neo-free progeny were back-crossed to C57BL/6n mice (Charles River Laboratories) to remove the *EIIA*-Cre cassette. Genotyping of neo-free mice was performed with primers P1, P4, and P2-625 (BGH-pA): 5′-CCA TCT GTT GTT TGC CCC TC-3′. Mice were backcrossed for 9 generations to C57BL/6n (Charles River Laboratories), and all experiments were performed with animals in the C57BL/6n genetic background.

### Bleomcyin challenge, hydroxyproline measurements, and lung mechanics.

Mice were lightly anesthetized with isoflurane and 50 μL of 1 U/kg, 2 U/kg, or 3 U/kg bleomycin sulfate (15 U, Hospira NDC 61703-332-18) or vehicle (50 μL of 0.9% saline) administered via oropharyngeal aspiration using a micropipette. Mice were hung vertically by the upper 2 incisors on a horizontally extended string; the tongue was extended, and liquid was placed onto the distal part of the oropharynx while the nose was gently closed. After 2–3 deep inhalations, mice were placed in the cage for recovery and monitored. At the end of the study, mice were euthanized and the left lung was inflated with paraformaldehyde as described below; right lung lobes were partitioned for biochemical analyses (right upper and accessory lobe was frozen for Western blotting, right middle lobe was hydrolyzed for hydroxyproline assay, and right lower lobe was frozen for RNA). Total lung collagen was measured by assaying lung hydroxyproline content after hydrolysis of the right middle lung lobe with 6N HCl exactly as previously described (45). Lung mechanics were assessed on anesthetized mice using a computerized flexiVent system (SCIREQ) exactly as previously described (45).

### Histology, immunofluorescence, and second harmonic imaging.

Lungs were inflated with 4% paraformaldehyde under 25 cm pressure and immersed in the same fixative overnight at 4°C. Right lung lobes and left lung were subdissected and embedded in paraffin after dehydration in an ethanol series. Lung pieces were sectioned at 5 μm using a Leica RM2235 microtome for H&E staining. Images in Supplemental Figure 1 were obtained at 10× with Zeiss Axio A2 microscope equipped with an AxioCam MRc5 camera. Tile scans of H&E-stained sections were obtained at 4× magnification using a Nikon NiE upright microscope system. Total area of the lung lobe and area of bleomycin-injured lung were calculated using Nikon Elements by drawing a user-defined region of interest (ROI). Percentage of lung injured was defined as (injured ROI/lung ROI) × 100. For immunofluorescence analyses, standard procedures were followed, and sections were stained with primary antibodies (Supplemental Table 1) with sodium citrate antigen retrieval as required. All Alexa Fluor–conjugated secondary antibodies (Supplemental Table 2) were used at a dilution of 1:200. High-magnification images were taken at 60× with Nyquist magnification or 100× using a Nikon A1 LUNA inverted microscope. For second harmonic imaging, 5 μm–thick paraffin sections were dewaxed in xylene, rehydrated in an ethanol gradient series, and placed in PBS. Tile scans at 10× magnification (air objective, plan apochromat) were obtained using Nikon A1R multiphoton upright microscope. Spectral unmixing was performed to separate the second harmonic signal for collagen and auto fluorescence of the lung tissue. Threshold function on general analysis program (Nikon Elements) was used to determine the area of signals from second harmonic and auto-fluorescence channels.

### Morphometric analyses.

Morphometric analyses for adult mice (P30 and older) were performed exactly as previously described (46). For neonatal mice, 5–8 random fields of immunofluorescent stained lung sections were imaged at 60× with Nyquist magnification or 100× using a Nikon A1 LUNA inverted microscope. Images contained at least 8 individual 1 μm optical sections. A binary channel for DAPI was created using general analysis program on Nikon Elements to obtain clear nuclear boundaries between cells in clusters. Processed images with a DAPI binary channel were rendered in 3D, and cells were manually counted on Imaris (Bitplane version 9.3.0). The binary channel for DAPI was masked with a surface and object was split with seed points (diameter: P1–P4 = 3.2 m, P7 = 3.5 μm, P14 = 3.8 μm, and P21 = 4 μm) to determine total nuclei per image field. A total of 1200–1500, 500–700, 400–650, 200–350, and 180–300 nuclei per mouse were counted for P1, P4, P7, P14, and P21 lungs, respectively. Orthogonal projections of confocal *Z*-stacks were analyzed manually to verify Ki67 positivity of AT2 cells.

### AT2 cell isolation and organoid assay.

Lungs were inflated with 1 mL, 2 mL, and 2.5 mL dispase (BD Biosciences, 354235) for P4 pups, P21 pups, and adult mice, respectively. Lungs were perfused with 0.9% saline prior to dispase instillation only in adult mice. Single cell suspensions were prepared in C-tubes (Miltenyi Biotec, 130-096-334) using the gentleMACS dissociator (Miltenyi Biotec) with 120 U/mL DNaseI (MilliporeSigma, D4527). Suspensions were filtered through 40 μm strainers and incubated in RBC lysis buffer (BioLegend, 420301) for 4 minutes at 4°C (RBC lysis was performed only for P4 and P21 cell suspensions). Cells were washed with complete RPMI (with 10% FBS) and resuspended in MACS buffer (1× PBS + 2 mM EDTA + 0.05% BSA) and incubated with the following anti-mouse biotinylated antibodies: CD45 (BioLegend, 103104, clone 30-F11), CD16/32 (BD Pharmingen, 553143, clone 2.462), CD31 (BioLegend, 102503, clone MEC13.3), CD90.2 (BioLegend, 105304, clone 30-H12), and Ter119 (BioLegend, 116203, clone Ter119). Suspensions were subsequently incubated with anti-biotin microbeads (Miltenyi Biotec, 130-097-046) and purified over a LS column (Miltenyi Biotec, 130-042-401) attached to a QuadroMACS separator (Miltenyi Biotec). Eluates from the column (CD45^–^CD16/32^–^CD31^–^CD90^–^Ter119^–^) were stored at –80°C as a dry pellet for Western blot analysis or processed immediately for RNA isolation and biochemical analyses. MACS isolation resulted in AT2 cell yields in the range of 5 *×* 10^6^ to 8 *×* 10^6^ cells/mouse with a purity of 80-90% (determined by flow cytometry).

For generation of organoids, WT mixed background mice (*n* = 6) were subjected to pneumonectomy, and fibroblasts were harvested 5 days post-surgery (47). Single cell suspensions were prepared with dispase and DNaseI as above. Cell suspensions were incubated with Fc receptor block in MACS buffer for 10 minutes, followed by incubation with PDGFRα (CD140a^+^) microbeads (Miltenyi Biotec, 130-101-502) for 15 minutes. Cells were purified over LS columns attached to a QuadroMACS magnet. CD140a^+^ fibroblasts were cocultured with P4 AT2 cells in a 10:1 ratio, combined with matrigel (Trevigen, 3445-005-01) in a 1:1 ratio and seeded on an air liquid interface transwell system in a 24-well plate (Corning, 3495). The cocultures were incubated in MTEC plus media (DMEM-Ham’s F-12 [Invitrogen 11330-032], HEPES, penicillin and streptomycin, fungizone, insulin [MilliporeSigma, I-6634], transferrin [MilliporeSigma, T1147], cholera toxin [MilliporeSigma, C8052], EGF [MilliporeSigma, E4127], and bovine pituitary extract [Invitrogen, 13028-014]) for 21 days. Rock inhibitor Y27632 (Enzo, ALX-270-333) was included in the medium for the first 2 days (48). ROCK inhibitor was removed after 48 hours of culture, and media was supplemented with or without 10 μM BHA (MilliporeSigma, B1253). Medium was changed every 48 hours, and fresh BHA was included at each change. Organoids were cultured for 3 weeks, fixed in 4% PFA, and processed for immunofluorescence analysis. Images were obtained on a Nikon A1R inverted microscope, and analysis of differentiation markers was completed using general analysis program on Nikon Elements.

### Characterization of organoid size.

Organoid formation was quantified for both number and size using the Cytation 5 primary cellular analysis function. The parameters for each brightfield image were set as follows: channel, bright-field; threshold, auto; background, light; minimum object size, 20 μm; maximum object size, 1000 μm; fill holes in mask; include primary edge objects; split touching objects. Size and circularity criteria were used to eliminate nonspheroidal objects in addition to objects smaller than the minimal size. Every image that was collected for each transwell of organoid culture was run through the analysis program with set parameters. The number of organoids between 20–500 μm as well as 500–1000 μm for each treatment group was input into Prism for further statistical analysis.

### Metabolic labeling, SDS PAGE, and Western blotting.

Lung slices (1 mm)were incubated for 1 hour in methionine- and cysteine-free DMEM (MilliporeSigma, 21013-024) supplemented with 5% dialyzed FBS (Thermo Fisher Scientific, 26400). Slices were pulse labeled with 0.5 mCi/mL [^35^S] methionine/cysteine (Perkin Elmer, NEG772002MC) for 30 minutes. Labeled slices were homogenized in 190 mM NaCl, 6 mM EDTA, 60 mM Tris, pH 7.4, and 4% SDS; proteins were precipitated by trichloroacetic acid and incorporated [^35^S] counts per minute (cpm) determined with a liquid scintillation counter (Perkin Elmer Tricarb, 2910TR). Samples containing equal cpm were immunoprecipitated with 5 μL of rabbit mature SP-C antibody (Seven Hills Bioreagents, 76694) immobilized on 30 μL of precleared recombinant Sepahrose G beads (Invitrogen, 101242) overnight. Beads were washed 4 times with 150 mM NaCl, 50 mM Tris, pH 7.5, 5 mM EDTA, 0.1% Triton X-100, and 0.02% SDS, followed by 2 washes with 150 mM NaCl, 50 mM Tris, pH 7.5, and 5 mM EDTA, and resuspended in 6× Laemmli sample buffer containing BME for SDS-PAGE (Sigma-Aldrich). Gels were fixed in methanol/acetic acid, dried, and exposed to a phosphor screen, which was imaged at various intervals to detect radioactivity using a Typhoon FLA 9500 system (GE Healthcare).

For SDS-PAGE/Western blotting, AT2 cells were harvested and lysed by sonication at a concentration of 1 × 10^6^ cells/80 μL in 1× PBS containing 1% mammalian protease inhibitor cocktail (Sigma-Aldrich, P8340) and 1× PhosSTOP (Sigma-Aldrich, 4906837001). Protein concentration in the supernatants was assessed with the Pierce Micro BCA kit (Thermo Fisher Scientific, 23235). Equal amounts of protein were separated on 10%–20% tris-tricine (Thermo Fisher Scientific, EC66252BOX; Figure 3A and Supplemental Figure 5A, Supplemental Figure 6, and Supplemental Figure 13, A and D) or 10%–20% tris-glycine gels (Thermo Fisher Scientific, XP10202BOX; Figure 3D and Supplemental Figure 11, B and C), under reducing electrophoretic conditions at 125V for 1.5 hours and transferred to 0.1 μm nitrocellulose membranes (GE Amersham, 10600000) at 180 mA for 1 hour using a semidry apparatus. Membranes were blocked in 5% nonfat dry milk and subsequently incubated with primary antibodies (Supplemental Table 1) overnight at 4°C. Membranes were subsequently washed with 1× TBS containing 0.05%–0.1% Tween-20, incubated with the appropriate HRP-conjugated secondary antibodies (Supplemental Table 2), developed using Immobilon forte Western HRP substrate (EMD Millipore, WBLUF0100) or Pierce SuperSignal West Pico PLUS Chemiluminescent Substrate (Thermo Fisher Scientific, 34580) and analyzed on a ChemiDoc Touch Imaging System (Bio-Rad). Membranes were stripped with Restore Western blot stripping buffer (Thermo Fisher Scientific, 21063) prior to reprobing. Total protein normalization was performed using the ImageLab software (Bio-Rad) with gels stained with Expedeon instant blue (MilliporeSigma, ISB1L) after transfer.

### RNA isolation and qPCR.

Total RNA was isolated from 1 × 10^6^ to 2 × 10^6^ AT2 cells using the Quick-RNA miniprep kit (Zymo Research, R1054), and cDNA was synthesized using iScript cDNA Synthesis Kit (Bio-Rad, 1708891). qPCR was performed with 20 ng of cDNA per reaction on QuantStudio 3 (Thermo Fisher Scientific) with Taqman assays (Integrated DNA Technologies) for mouse *Sftpc* (IDT Mm.PT.58.9922071, exons 1–2), and *Bax* (IDT Mm.PT.58.14012210, exons 3–4). Multiple housekeeping genes were used to identify stable expression across developmental ages (P4 and P21), and data were normalized to mouse *Ppia* (IDT Mm.PT.39a.2.gs).

### scRNAseq and data analysis.

P4 and P21 lungs were inflated with 1 mL dispase, resected, and incubated in dispase for 6 minutes at 37°C. Lung lobes were separated and placed in C-tubes containing 5 mL RPMI (Thermo Fisher Scientific, 11875-093) supplemented with 10% FBS (MilliporeSigma, F4135), 1M HEPES, and 1% penicillin/streptomycin. Single cell suspensions were prepared using the gentleMACS dissociator with 120 U/mL DNaseI and filtered through a 40 μm strainer, washed with media, and incubated in RBC lysis buffer for 4 minutes at 4°C. Suspensions were washed with media, cell counts were performed, and cells were resuspended in RPMI at a final concentration of 100 cells/μL. GEM generation and barcoding, as well as cDNA library preparation, were completed by the CCHMC Gene Expression Core using the Chromium Single Cell 3′ Reagent Kit (10X Genomics version 2.0 and version 3.0). Sequencing was performed on NovaSeq 6000 at a depth of approximately 300–450 million reads. Read alignment and gene-expression quantification of data were performed using the CellRanger pipeline (10X Genomics version 2.0.0 and version 3.0.2). Cell Ranger R kit version 2.0.0 (10X Genomics) was used to process raw sequencing data and paired-end sequence alignment to mouse genome (mm10). Cells with at least 500 expressed genes (unique molecular identifier [UMI] > 0) and less than 10% of UMIs mapping to mitochondrial genes were included for downstream analysis. Genes expressed in at least 2 cells in each data set were included. Data sets were integrated with Harmony (49), and downstream analyses were performed in R (version 3.5.0) using custom scripts, SINCERA (50, 51), and Seurat (version 2.3.4) (52, 53). The expression of a gene in a cell was measured by its UMI counts in the cell normalized by the total number of UMIs in the cell. Principal component analysis was performed for dimension reduction. Reduced dimensions were used for cell cluster identification using the Jaccard-Louvain clustering algorithm (54). Clusters were mapped to cell types based on the expression of known marker genes. Cluster-specific differentially expressed genes were identified using a binomial based differential expression test implemented in the SINCERA pipeline (49, 50). Genes with *P* < 0.05 and effect size > 2 expressed in > 20% of the cells in a given cluster were considered significant, and functional enrichment analyses were performed using Toppfun in ToppGene suite (55). All Venn diagrams were generated on BioVenn (56).

### Measurement of ROS.

ROS levels were measured with the Abcam DCFDA/H2DCFDA cellular ROS assay kit (ab113851) according to manufacturer’s instructions. Briefly, 1 *×* 10^6^ freshly isolated AT2 cells were stained with 20 μM DCFDA for 30 minutes at 37°C. Approximately 2 *×* 10^5^ cells/well were seeded in a 96-well microplate, and fluorescence was immediately measured with the Synergy2 Multimode Microplate Reader (BioTek) with excitation/emission wavelengths of 485/535 nm. Cells were treated with 50 μM TBHP for induction of oxidative stress.

### Measurement of glutathione concentrations.

Glutathione concentrations were measured with the Invitrogen Glutathione Colorimetric Detection Kit (Thermo Fisher Scientific, EIAGSHC) per manufacturer’s instructions. Briefly, 4 *×* 10^6^ freshly isolated AT2 cells were deproteinized with 500 μL 5% salicylic acid (Sigma-Aldrich, S2130). Lysates were centrifuged at 16,000*g* for 10 minutes at 4°C, and the clarified supernatant was divided in equal amounts for measurement of GSH (reduced glutathione) and GSSG (oxidized glutathione); supernatant was incubated with 2-vinylpyridine (Sigma-Aldrich, 132292) for 1 hour at room temperature prior to measurement of GSSG. Absorbance of standards and samples was measured at 405 nm with the Synergy2 Multimode Microplate Reader (BioTek).

### Proteomics and data analysis.

AT2 cells were isolated as described above, and dried frozen cell pellets were submitted to MS Bioworks for 6-plex tandem mass tags (TMT) proteomics and data analysis. Briefly, cells were lysed with 150 μL of urea lysis buffer (8M urea, 50 mM Tris-HCl, pH 8, 150 mM NaCl). Cell lysates were centrifuged at 10,000*g* for 10 minutes at 4°C, and clarified supernatant was used for protein quantitation using a Qubit protein assay (Invitrogen). Samples were digested and processed by solid phase extraction followed by addition of the TMT label (Thermo Fisher Scientific, 90110). Labeled samples were extracted and fractionated using an Agilent 1100 HPLC system. Sample fractions were analyzed by nano LC-MS/MS with a Waters M-class LC system interfaced to a Thermo Fisher Scientific Fusion Lumos. Data were processed with MaxQuant (version 1.6.14.0) and a 2-tailed *t* test was performed to determine significance for reporter ion intensities. A total of 6434 proteins was identified and abundance of 1117 proteins was deemed as significant based on a cut-off of *P* < 0.05 (Supplemental Dataset File). A heatmap was generated using Heatmapper (57), and gene ontology and pathway analyses for significantly abundant proteins were performed using Toppfun in the Toppgene suite (55).

### Data availability.

scRNAseq data are available on GEO (accession no. GSE155814; https://www.ncbi.nlm.nih.gov/geo/query/acc.cgi?acc=GSE155814). Proteomics data are available on the MassIVE data repository (ID-MSV000085976; https://massive.ucsd.edu/ProteoSAFe/dataset.jsp?accession=MSV000085976).

### Statistics.

Statistical analysis was performed using GraphPad Prism (GraphPad Software). All data are presented as mean ± SD. Statistical significance was determined by unpaired 2-tailed *t* tests or 1- or 2-way ANOVA. A *P* value less than 0.05 was considered significant.

### Study approval.

Mice were housed in a pathogen-free barrier facility. All animal procedures were performed under protocols (IACUC2015-0073) approved by the IACUC of Cincinnati Children’s Hospital Medical Center.

## Author contributions

SS and TEW developed concepts, interpreted data, and prepared the manuscript. SS, EPM, CLN, SZ, JG, HD, AKTP, JPB, and YX conducted experiments and analyzed data. All authors contributed to the data interpretation and manuscript editing. All the authors read and approved the final manuscript.

## Supplementary Material

Supplemental data

Supplemental Data Set 1

## Version 1 06/17/2021

In-Press Preview

## Version 2 07/22/2021

Electronic publication
