# Supplementary material for: Surfactant protein C mutation links postnatal type 2 cell dysfunction to adult disease
Source: JCI Insight. 2021 Jul 22;6(14):e142501. doi: 10.1172/jci.insight.142501 (PMC8410047; doi:10.1172/jci.insight.142501)
Supplement: Supplemental data [file jciinsight-6-142501-s162.pdf]

Supplemental Information

Surfactant protein C mutation links postnatal type 2 cell dysfunction to adult disease

Sneha Sitaraman, Emily P. Martin, Cheng-Lun Na, Shuyang Zhao, Jenna Green, Hitesh Deshmukh, Anne-Karina T. Perl, James P. Bridges, Yan Xu, Timothy E. Weaver

Methods.

Collection of bronchoalveolar lavage fluid (BALF), cell counts and measurement of surfactant lipids.

BALF was collected by intratracheal intubation and 5 serial lung lavages with 1 ml 0.9% saline. Immune cells were isolated from BALF by centrifugation at 230 g for 10 minutes and pellets were resuspended in 100 µl of 0.9% saline for total cell counts. Cell-free BALF was used for measurement of surfactant lipids. Lipids were extracted from cell-free BALF by the Bligh and Dyer method (1). Saturated phosphatidylcholine was isolated using the osmium tetroxide-based method of Mason et al (2) and quantitated by phosphorous measurement.

Measurement of global protein synthesis.

AT2 cells were isolated as described above. 1 x 10^6 cells were incubated at 37°C for 40 minutes in complete RPMI medium lacking methionine (Invitrogen A1451701), supplemented with 10% dialyzed FBS, 25 mM HEPES, 1 mM sodium pyruvate and 2 mM L-glutamine. Cells were labeled for 160 minutes at 37°C with 500 µM L-Azidohomoalanine (L-AHA; Invitrogen C10102) in complete RPMI medium lacking methionine. Labeled cells were washed with 1X PBS, pooled to a total of 4 x 10^6 cells per tube and frozen at -80°C. Cell lysates were prepared by sonication in 200 µl lysis Buffer (50 mM Tris-HCl, pH 8, 1% SDS) and total protein was quantitated using the Pierce Micro BCA kit. Samples were biotinylated using the Click-iT Protein Reaction Buffer Kit (Invitrogen C10276); the click reaction was completed using 100 µg L-AHA-labeled protein in combination with or without biotin-alkyne (Invitrogen B10185), according to manufacturer's protocol. Samples were immediately precipitated using methanol and chloroform and stored at -20°C. Dried pellets were re-solubilized using 100 µl of lysis Buffer (50 mM Tris-HCl, pH 8; 1% SDS), followed by heating at 70°C for 10 minutes with gentle mixing by vortex. 20 µl of the non-reduced sample was electrophoresed on a 10-20% tris-tricine gel and transferred to 0.1 µm nitrocellulose membrane, followed by blocking in 5% NFDM for 1 hour at room temperature. Membranes were incubated for 1 hour at 4°C with anti-biotin-HRP antibody (Cell Signaling 7075; 1:1000 in 5% NFDM). Blots were developed with Pierce SuperSignal West Pico PLUS Chemiluminescent Substrate and imaged using the ChemiDoc

system. Results were normalized to total protein detected in gels stained with Instant Blue post transfer using the Biorad ImageLab software.

Flow cytometric analyses of alveolar macrophages

Lung tissues from 2-3 newborn mice were pooled together, diced and incubated at 37 °C for 30 minutes in a rotating incubator (150 rpm) in digestion buffer [RPMI 1640, 10% FBS, 15 mM HEPES, 1% penicillin/streptomycin (wt/vol) and 300 U/ml collagenase VIII]. Lung pieces were passed through a 100-μm nylon strainer and centrifuged at 400 g for 5 minutes at 4°C. 1 x 10^7 cells were then incubated with Zombie-Green dye (diluted 1:1000; BioLegend 423105) and anti-mouse CD16/CD32 to block Fc receptors for 30 minutes at 4°C. Cells were re-incubated with anti–mouse CD11b antibody (M1/70), anti-mouse CD11c antibody (N418), anti-mouse F4/80 antibody (BM8) and anti-mouse MHC II antibody (M5/114.15.2) [all diluted 1:100; BioLegend] for 30 minutes at 4°C. Surface-stained cells were washed, fixed and permeabilized in 1X True Nuclear Transcription Factor buffer (BioLegend 424401) containing anti–mouse CD163 antibody (S150491), anti-mouse ARG1 antibody (A1exF5) and anti-mouse CD206 antibody (C086C2) (all diluted 1:100, BioLegend) for 60 minutes at 4°C. Subsequently, cells were washed twice and resuspended in FACS buffer (1X PBS, 5% FBS, 2 mM EDTA). Data were acquired on the LSRII flow cytometer (BD Biosciences) and analyzed with FlowJo software (version 9.1; Treestar). Alveolar macrophages were identified as live $MHCII^+$ $F4/80^+$ $CD11c^+$ $CD11b^-$ cells.

References.

1. Bligh EG, and Dyer WJ. A rapid method of total lipid extraction and purification. *Can J Biochem Physiol.* 1959;37(8):911-7.
2. Mason RJ, Nellenbogen J, and Clements JA. Isolation of disaturated phosphatidylcholine with osmium tetroxide. *J Lipid Res.* 1976;17(3):281-4.

Supplemental figure 1

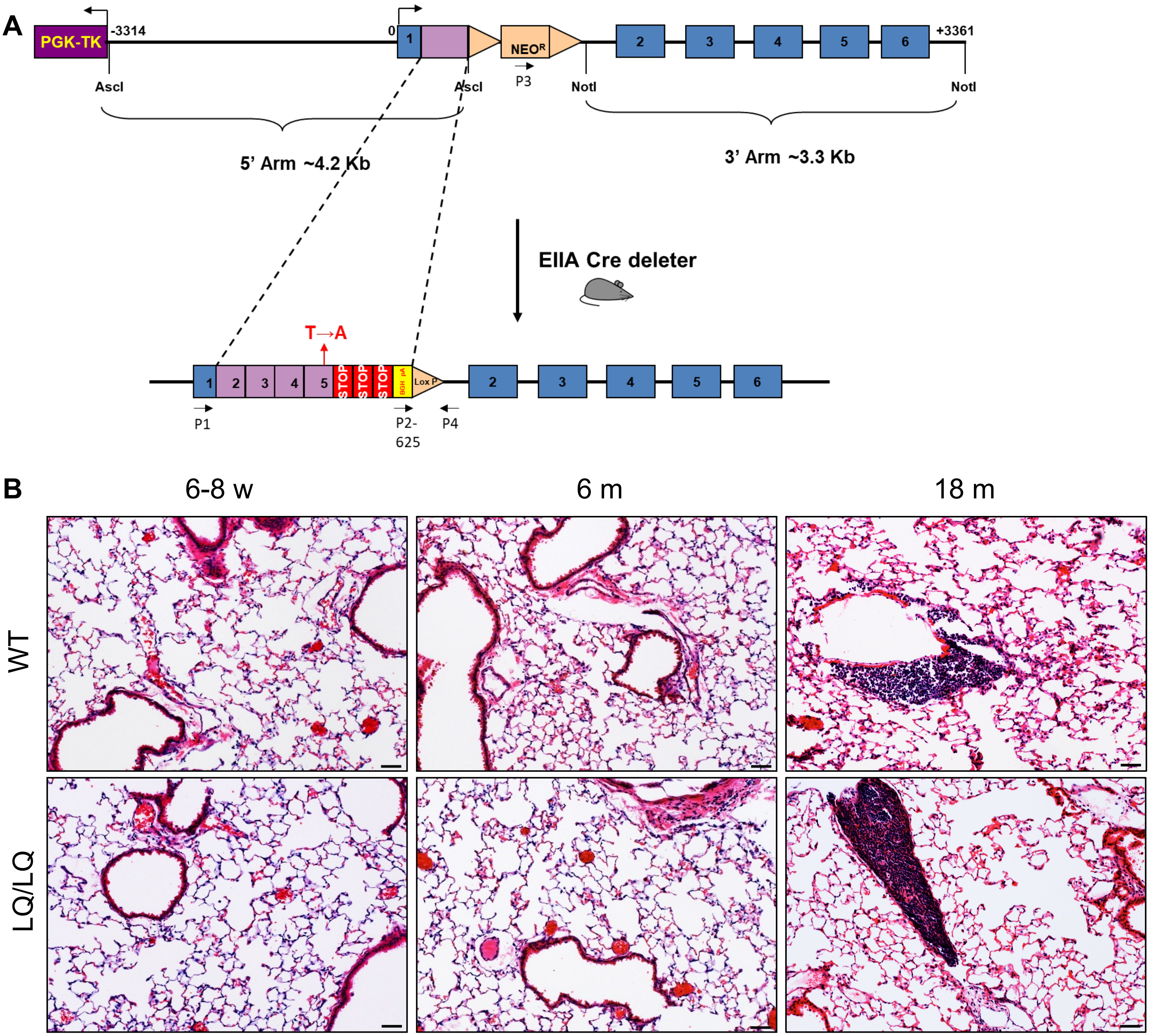

Figure 1: Expression of proSP-C^{LQ} does not disrupt lung structure in adult mice.
A. Knock-in cassette design. Founder mice carrying the knock-in allele were crossed to *EIIA*-Cre deleter mice to excise the neomycin resistance cassette. P1, P2-625, P3, P4: location of primers for genotyping. **B.** Representative H&E images of lung sections obtained from LQ/LQ mice. Note that perivascular lymphocytic infiltration observed in 18-month-old mice is an age-related phenotype also observed in WT mice. n = 3-6 mice/timepoint. Scale bars = 50 µm.

Supplemental figure 2

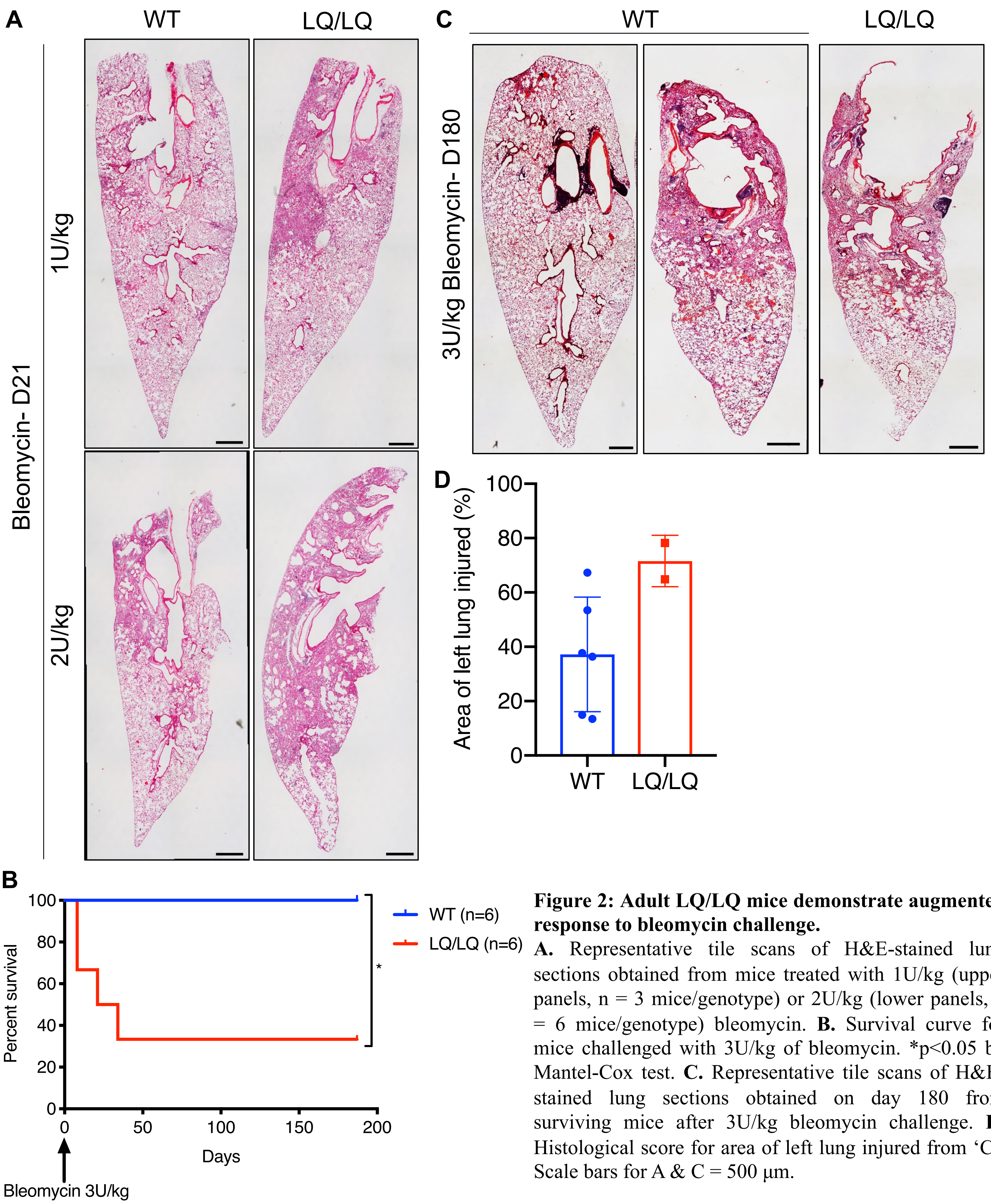

Figure 2: Adult LQ/LQ mice demonstrate augmented response to bleomycin challenge.
A. Representative tile scans of H&E-stained lung sections obtained from mice treated with 1U/kg (upper panels, n = 3 mice/genotype) or 2U/kg (lower panels, n = 6 mice/genotype) bleomycin. **B.** Survival curve for mice challenged with 3U/kg of bleomycin. *$p<0.05$ by Mantel-Cox test. **C.** Representative tile scans of H&E-stained lung sections obtained on day 180 from surviving mice after 3U/kg bleomycin challenge. **D.** Histological score for area of left lung injured from 'C'. Scale bars for A & C = 500 μm.

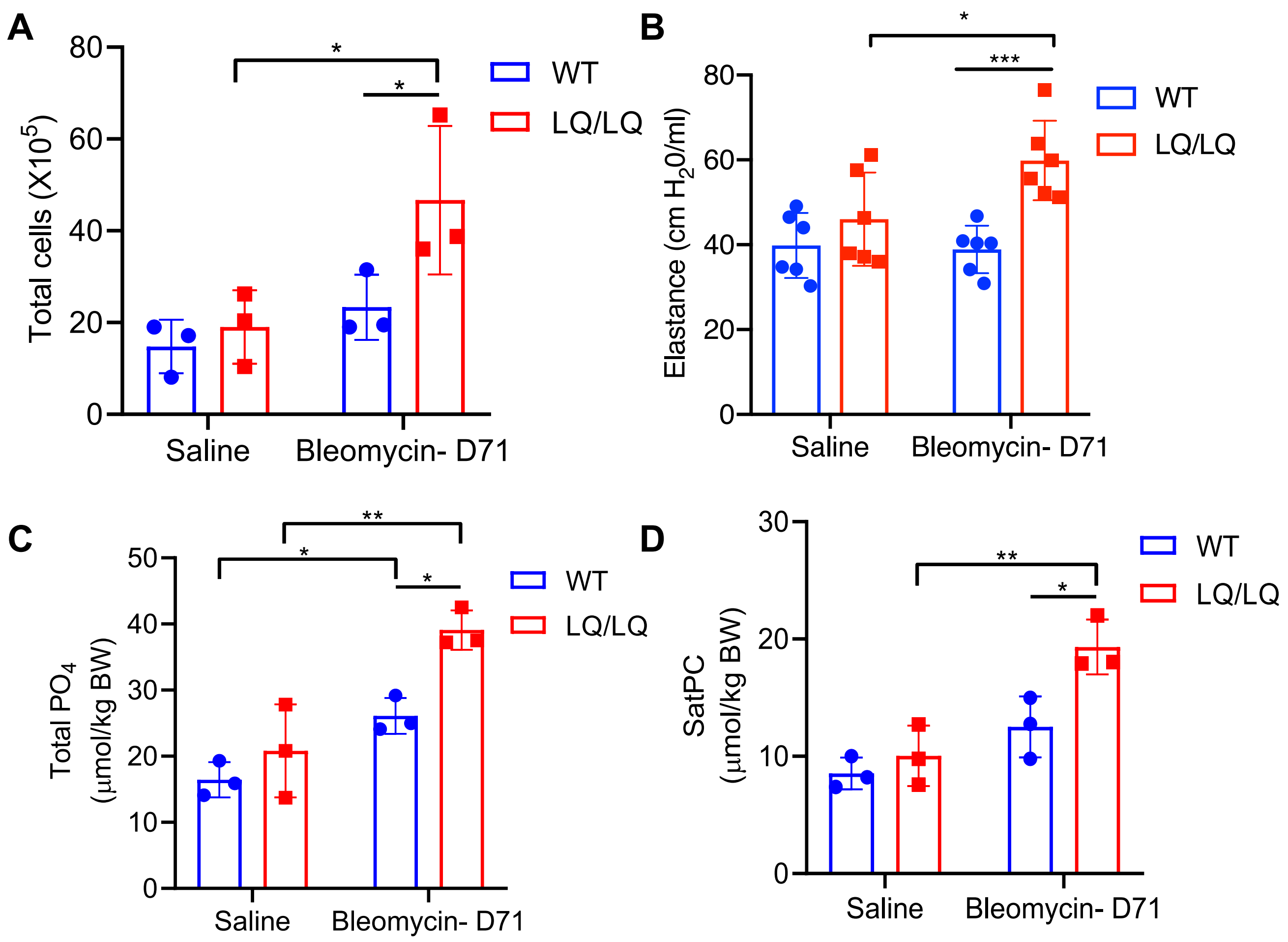

Figure 3: Persistent fibrotic response in adult LQ/LQ mice.
A. Total number of immune cells recovered from bronchoalveolar lavage fluid (BALF). **B.** Lung tissue elastance measured using FlexiVent. **C-D.** Concentration of total phosphate (PO_4) and saturated phosphatidylcholine (SatPC) in cell-free BALF, normalized to body weight (BW). *$p<0.05$, **$p<0.01$, ***$p<0.001$ by two-way ANOVA with Sidak's multiple comparison test.

Supplemental figure 4

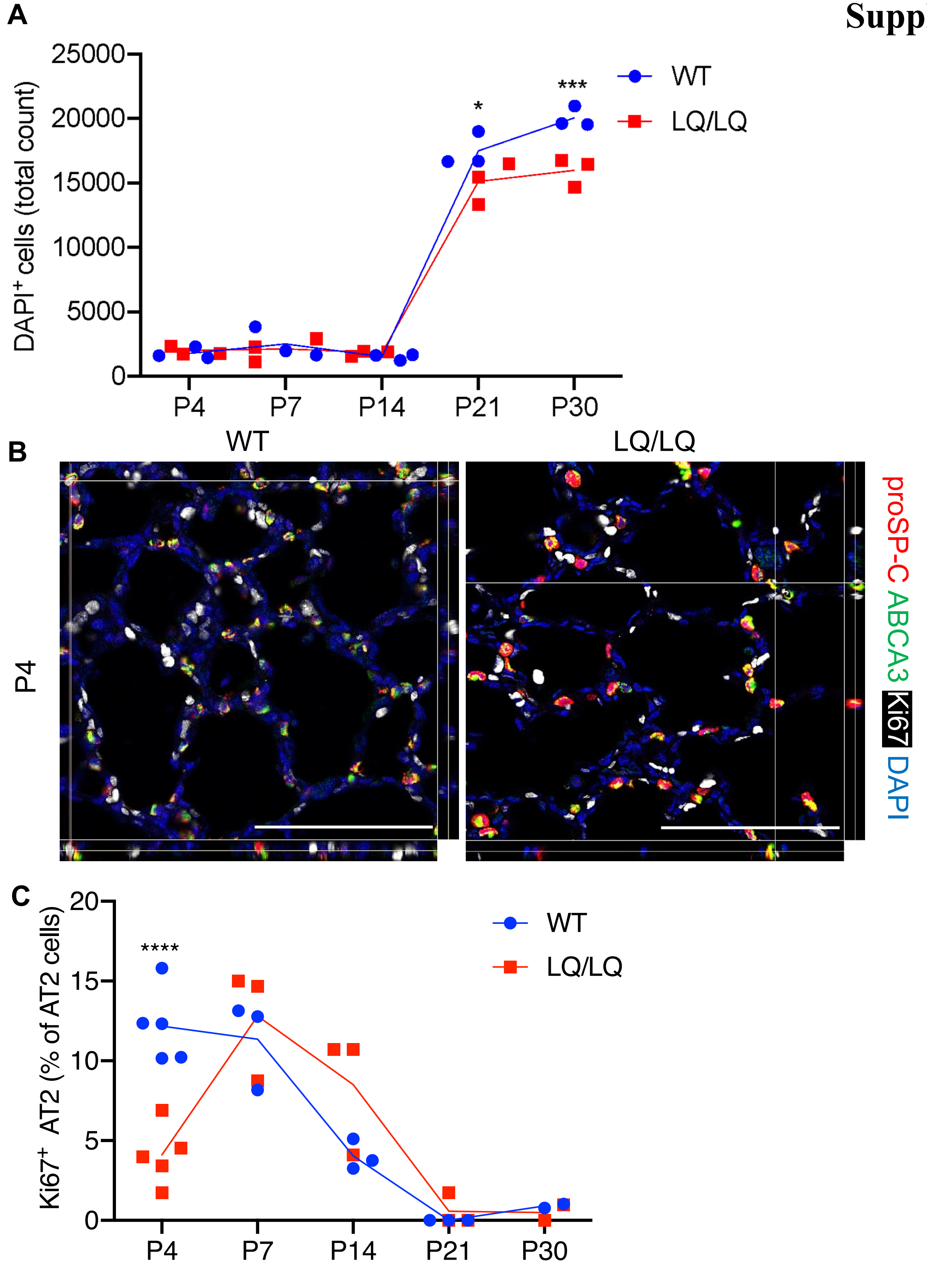

Figure 4: Expansion of neonatal AT2 cells is impaired in LQ/LQ mice.
A. DAPI counts obtained from morphometric analyses shown in figure 2D. **B.** Representative orthogonal views of confocal imaged P4 lung sections stained with proSP-C, ABCA3 and Ki67. Lines intersect at a proSP-C$^+$ ABCA3$^+$ Ki67$^+$ AT2 cell. Scale bars = 50 µm. **C.** Morphometric analyses of lung sections stained with proSP-C, ABCA3 and Ki67. Cell counts were obtained from confocal z-stacks rendered in 3D. Ki67 positivity for proSP-C$^+$ or ABCA3$^+$ cell was verified from xz and yz views. ****p<0.0001 by one-way ANOVA with Tukey's multiple comparison test.

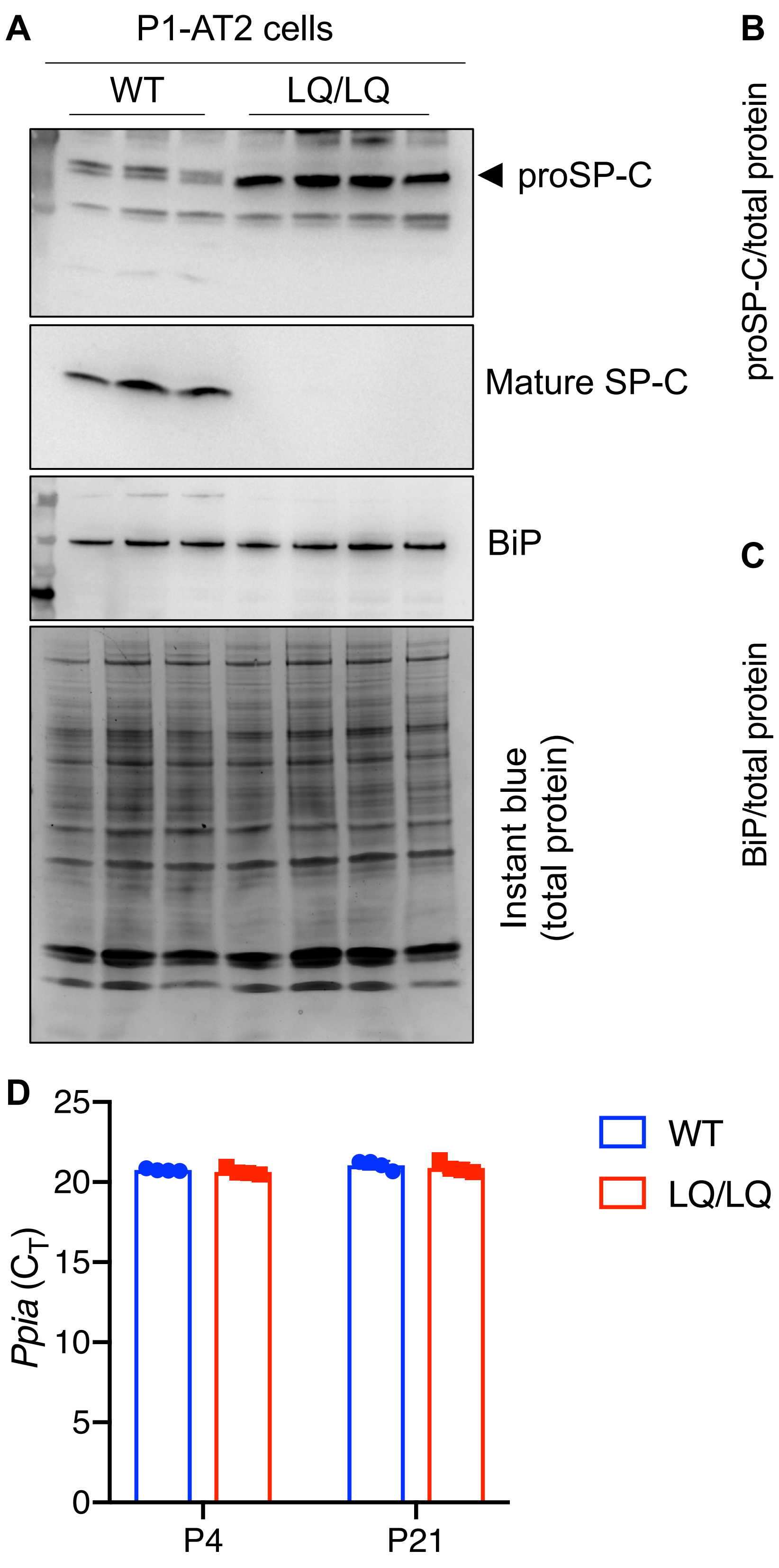

Figure 5: Accumulation of proSP-C is observed in P1 LQ/LQ AT2 cells.
A. Western blot analyses of 18 µg of P1 AT2 cell lysates separated by SDS-PAGE. Gel was stained with Coomassie-based instant blue to assess protein loading. Arrowhead points to SP-C proprotein. **B-C.** proSP-C (B) and BiP (C) levels from 'A' normalized to total protein. **D.** Cycle threshold (C_T) for *Ppia* used as housekeeping gene for quantitative real time PCR in figures 3C & 6F.

Supplemental figure 6

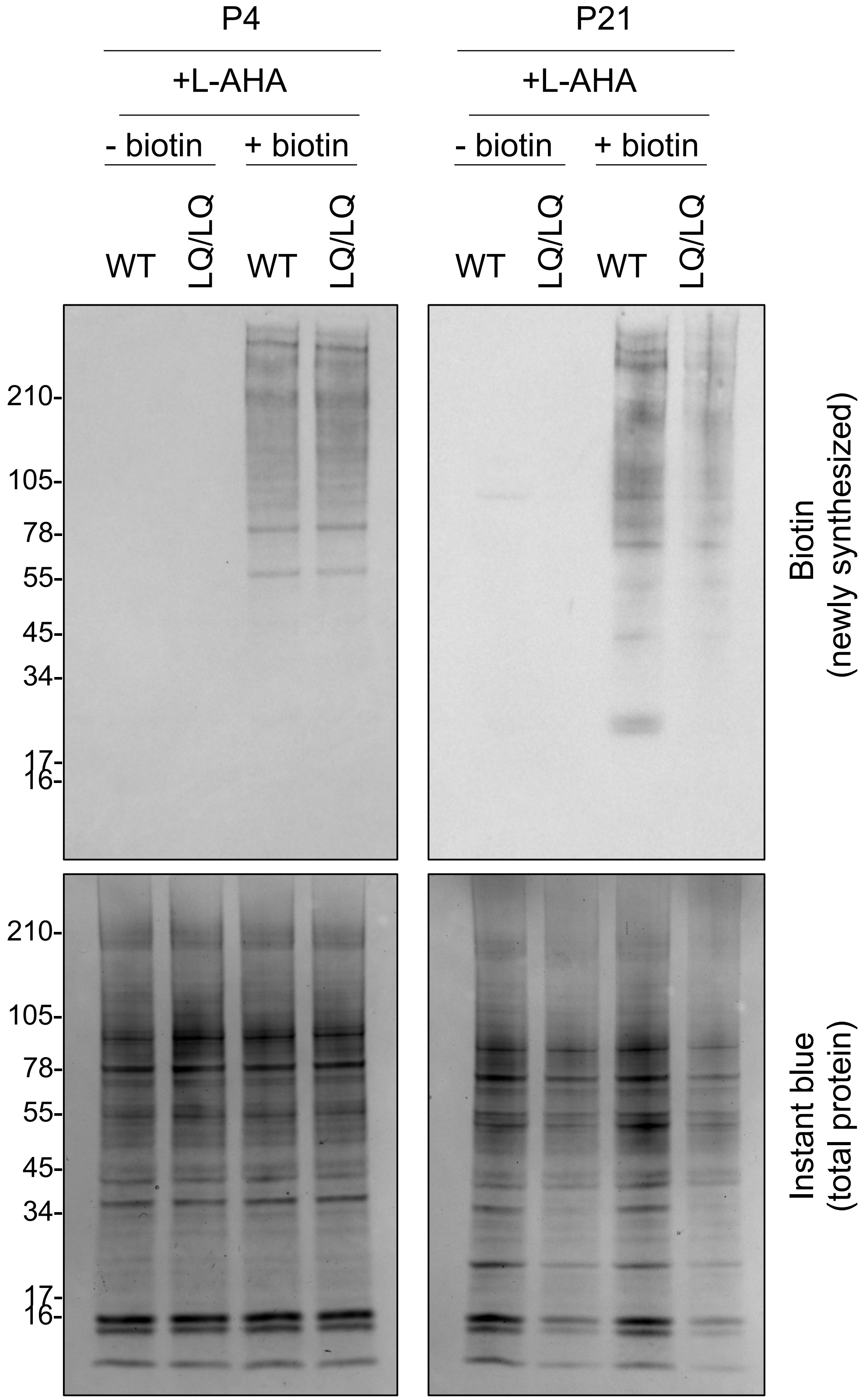

Figure 6: Global protein synthesis in P4 and P21 LQ/LQ AT2 cells.
Freshly isolated AT2 cells were labelled with amino acid analog L-AHA followed by ligation to biotin alkyne by 'click' reaction. Western blot analyses for biotinylated proteins from labelled AT2 cell lysates separated by SDS-PAGE shows newly synthesized proteins. Gels were stained with Coomassie-based instant blue to assess protein loading. '- biotin' lane indicates AHA labelled AT2 cell lysates without biotin alkyne ligation.

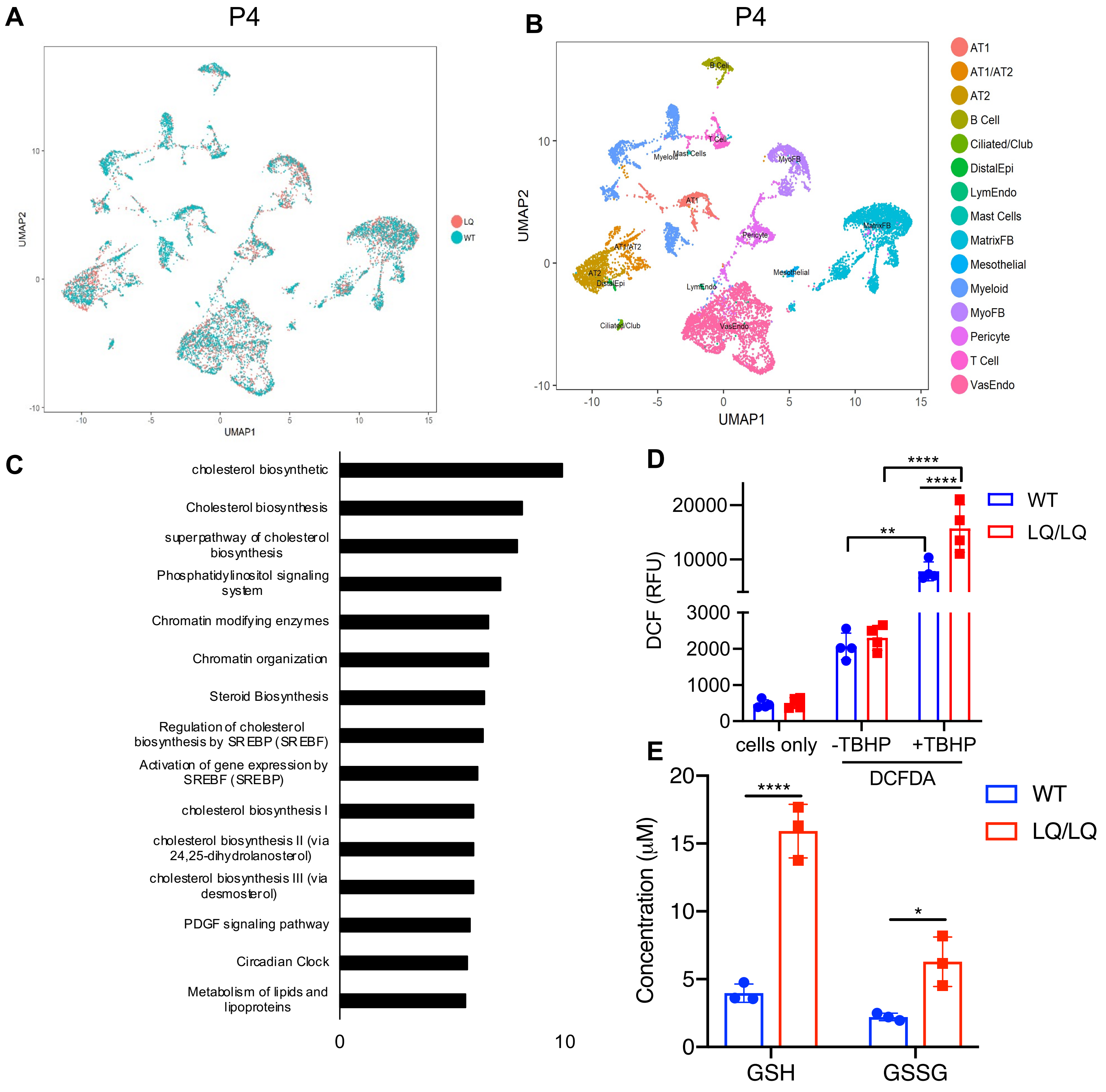

Figure 7: Activation and resolution of oxidative stress in LQ/LQ AT2 cells.
A. UMAP embedding shows populations from scRNAseq of P4 lungs identified by Cell Ranger. WT and LQ/LQ datasets were integrated using Harmony. **B.** Major populations identified in P4 WT and LQ/LQ datasets by Cell Ranger. **C.** Toppfun analyses of pathways characterizing the P4 WT AT2 cell population compared to LQ/LQ. Pathways are ordered by descending p-values (X-axis: -log10 p-value). **D.** Freshly isolated P21 AT2 cells were incubated with DCFDA for measurement of ROS levels. Cells without DCFDA were used as control to measure background fluorescence. RFU: relative fluorescence unit, TBHP: tert-butyl hydrogen peroxide (inducer of oxidative stress). **E.** Glutathione (GSH) concentrations were measured in freshly isolated, deproteinized P21 AT2 cells. AT2 cell lysates were incubated with 2-vinylpyridine for measurement of oxidized glutathione (GSSG). For D & E, $*p<0.05$, $**p<0.01$, $****p<0.0001$ by two-way ANOVA with Sidak's multiple comparison test.

Supplemental figure 8

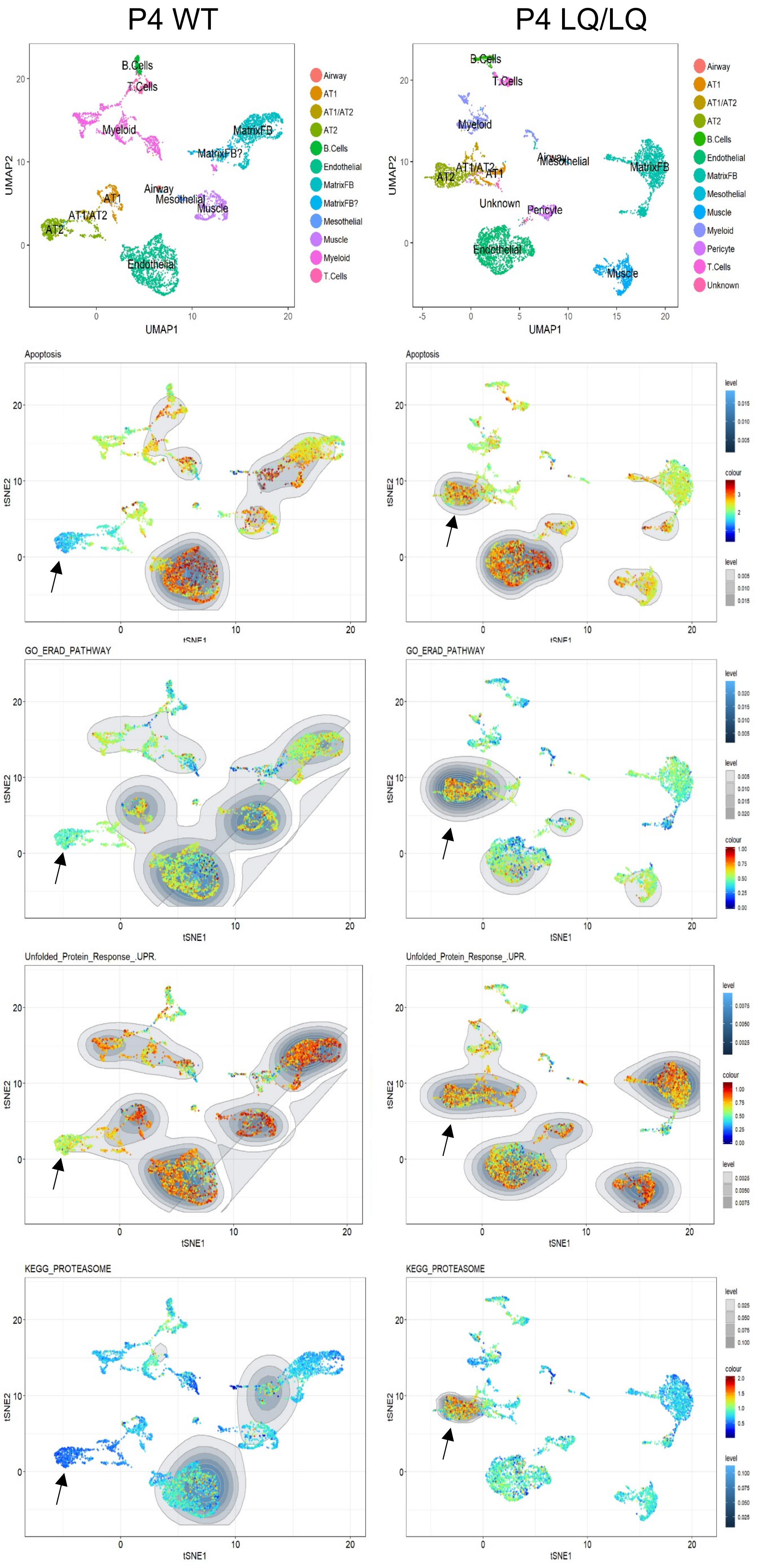

Figure 8: Activation of the ER associated degradation (ERAD) pathway in P4 LQ/LQ AT2 cells. UMAP embedding shows cell populations from scRNAseq of P4 WT and LQ/LQ lungs. Single cell explorer shows expression level of genes associated with apoptosis, ERAD, unfolded protein response and the proteasome pathways across all populations obtained from scRNAseq of P4 lungs. Arrows point to AT2 cells.

Supplemental figure 9

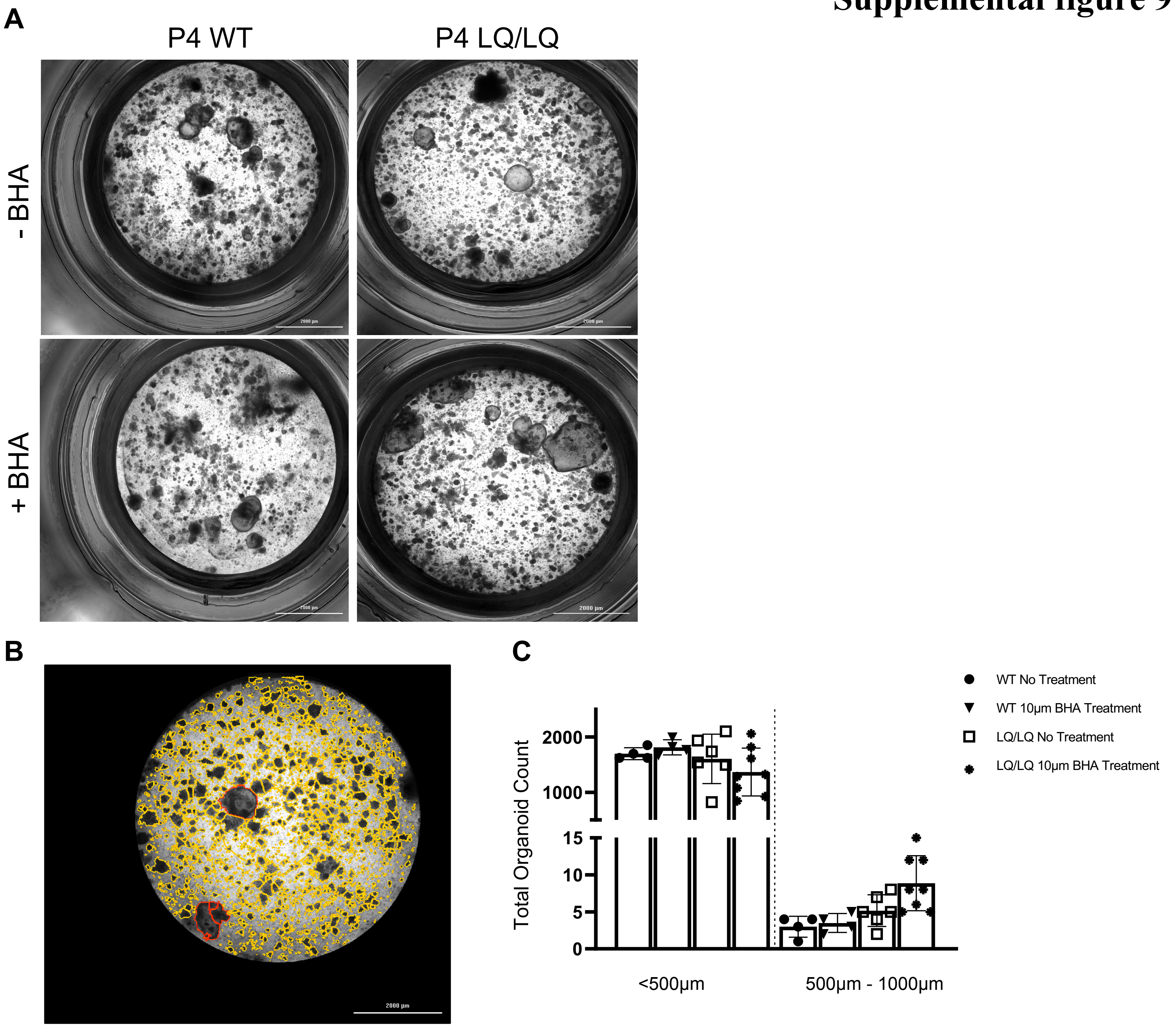

Figure 9: Colony forming efficiency is unchanged between WT and LQ/LQ organoids
A. Brightfield images of organoids derived from AT2 cells isolated from P4 WT and LQ/LQ lungs. Organoids were treated with or without the antioxidant, butylated hydroxyanisole (BHA). **B.** Quantitation of organoid colony sizes using Cytation 5 primary cellular analysis function. **C.** Number of organoid colonies delineated by size. Scale bars in B-C = 2000 μm.

Supplemental figure 10

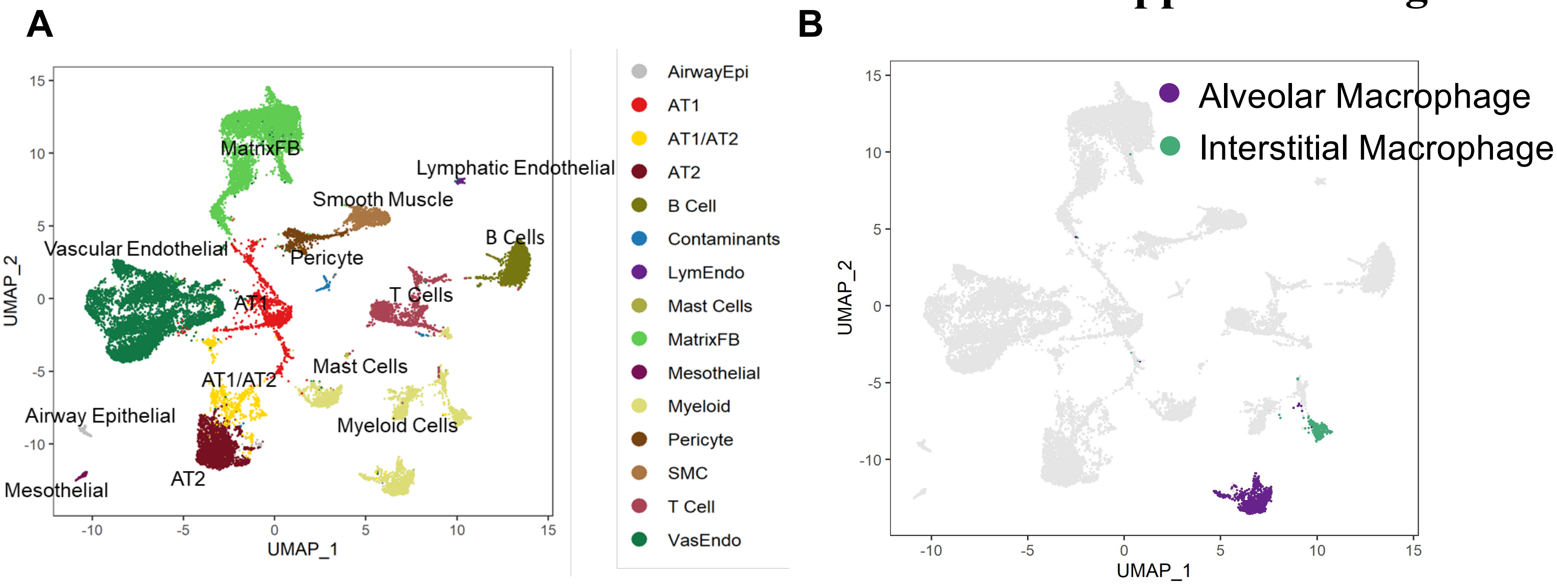

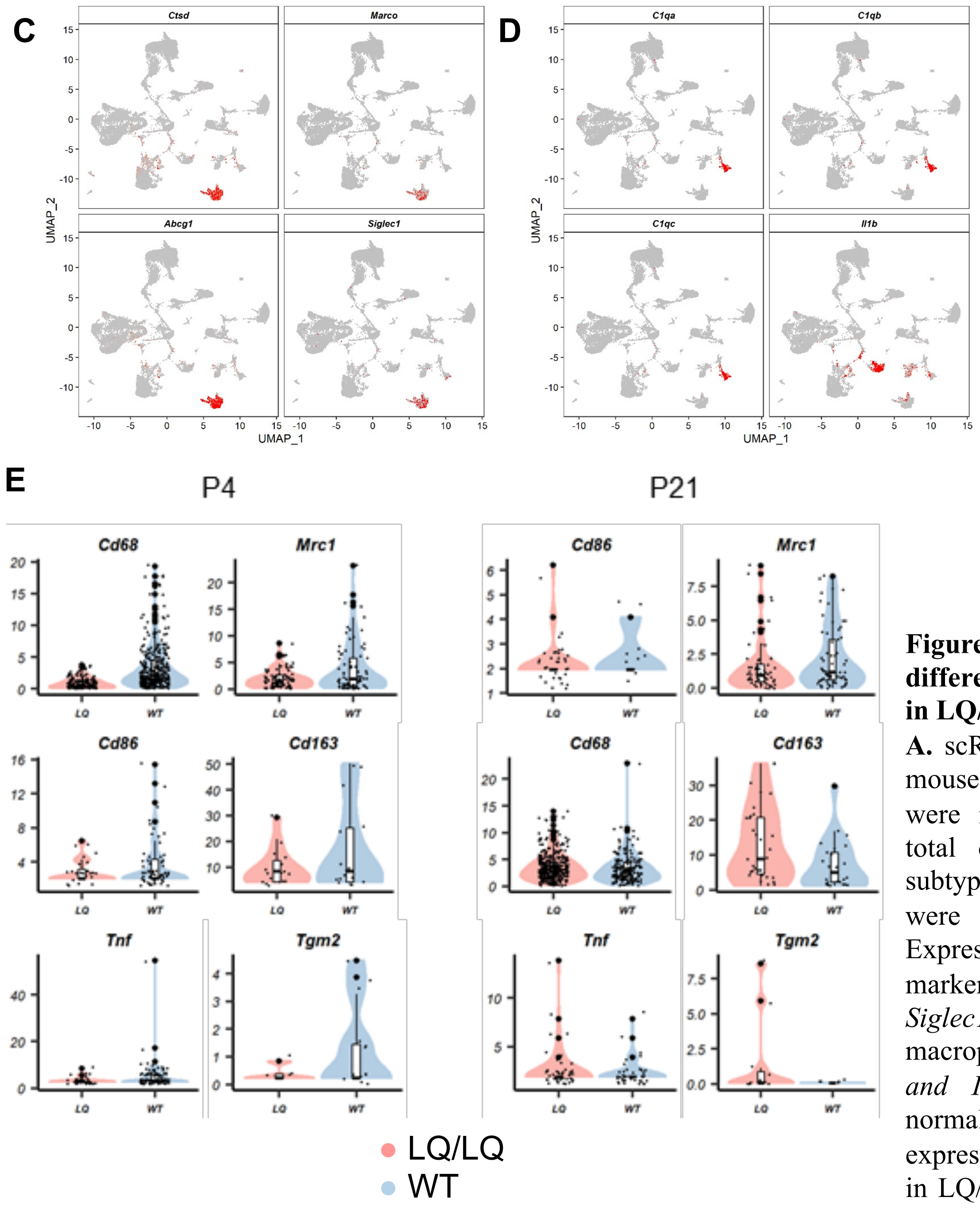

Figure 10: Decreased expression of differentiation markers in macrophages in LQ/LQ lungs.
A. scRNAseq data from LQ/LQ and WT mouse lung samples from P4 and P21 were integrated using Harmony with a total of 25,362 cells. **B.** Macrophage subtypes were identified, and the cells were visualized using UMAP. **C.** Expression of alveolar macrophage markers (*Ctsd, Marco, Abcg1, and Siglec1*). **D.** Expression of Interstitial macrophage markers (*C1qa, C1qb, C1qc, and Il1b*). The expression is *z*-score normalized. **E.** Violin plots show the expressions of select macrophage markers in LQ/LQ and WT lungs for both P4 and P21. The expression is z-score normalized.

Supplemental figure 11

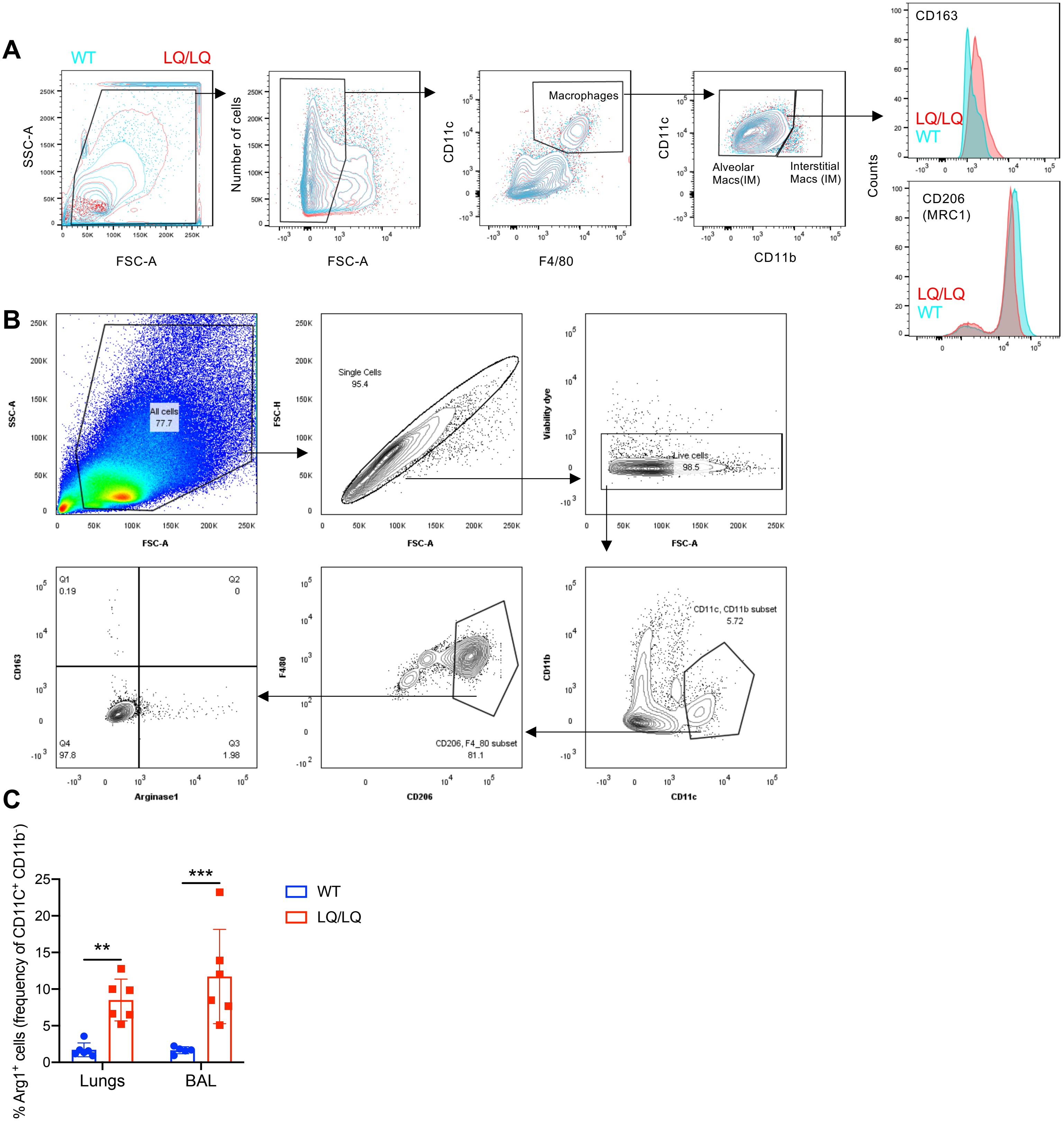

Figure 11: Inflammatory response in LQ/LQ lungs.

A. Gating strategy to identify pulmonary macrophage subsets in WT or LQ/LQ P14 mice. Alveolar macrophages were identified as live F4/80$^+$ CD11c$^+$ CD11b$^-$ cells. Interstitial macrophages were identified as live F4/80$^+$ CD11c$^+$CD11b$^+$ cells. Histograms showing expression of CD163 or CD206 (encoded by MRC1) on alveolar macrophages. Representative bivariate plots of single cell suspension from WT or LQ/LQ lung samples. **B.** Gating strategy to identify M2 polarized alveolar macrophages (CD11c$^+$ CD11b$^-$ F4/80$^+$ CD206$^+$). **C.** Frequency of Arginase1$^+$ (Arg1; M2 macrophage marker) alveolar macrophages in single cell suspensions of lungs and brochoalveolar lavage (BAL) fluid. **p<0.01, ***p<0.001 by two-way ANOVA with Sidak's multiple comparison test.

Supplemental figure 12

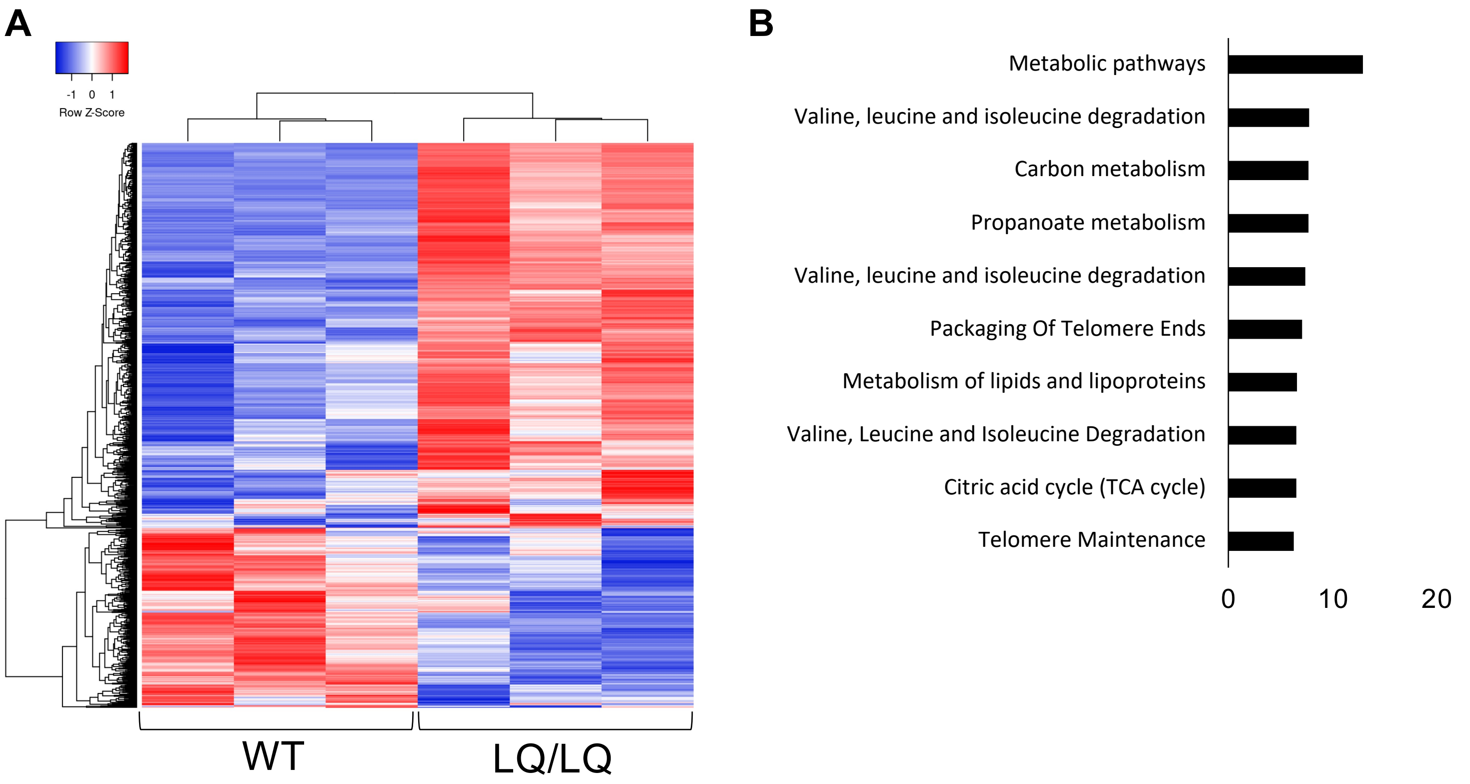

Figure 12: Proteomic analyses of P4 WT and LQ/LQ AT2 cells.
A. Heatmap shows protein abundance in P4 AT2 cells. Significance of protein abundance was determined by t-test and p-value <0.05 was reported as significant. Heatmap is z-score normalized. **B.** Toppfun analyses of proteomics data shows under-represented pathways in P4 LQ/LQ AT2 cells compared to P4 WT AT2 cells. Pathways are ordered by descending p-values (X-axis: $-\log_{10}$ p-value).

Supplemental figure 13

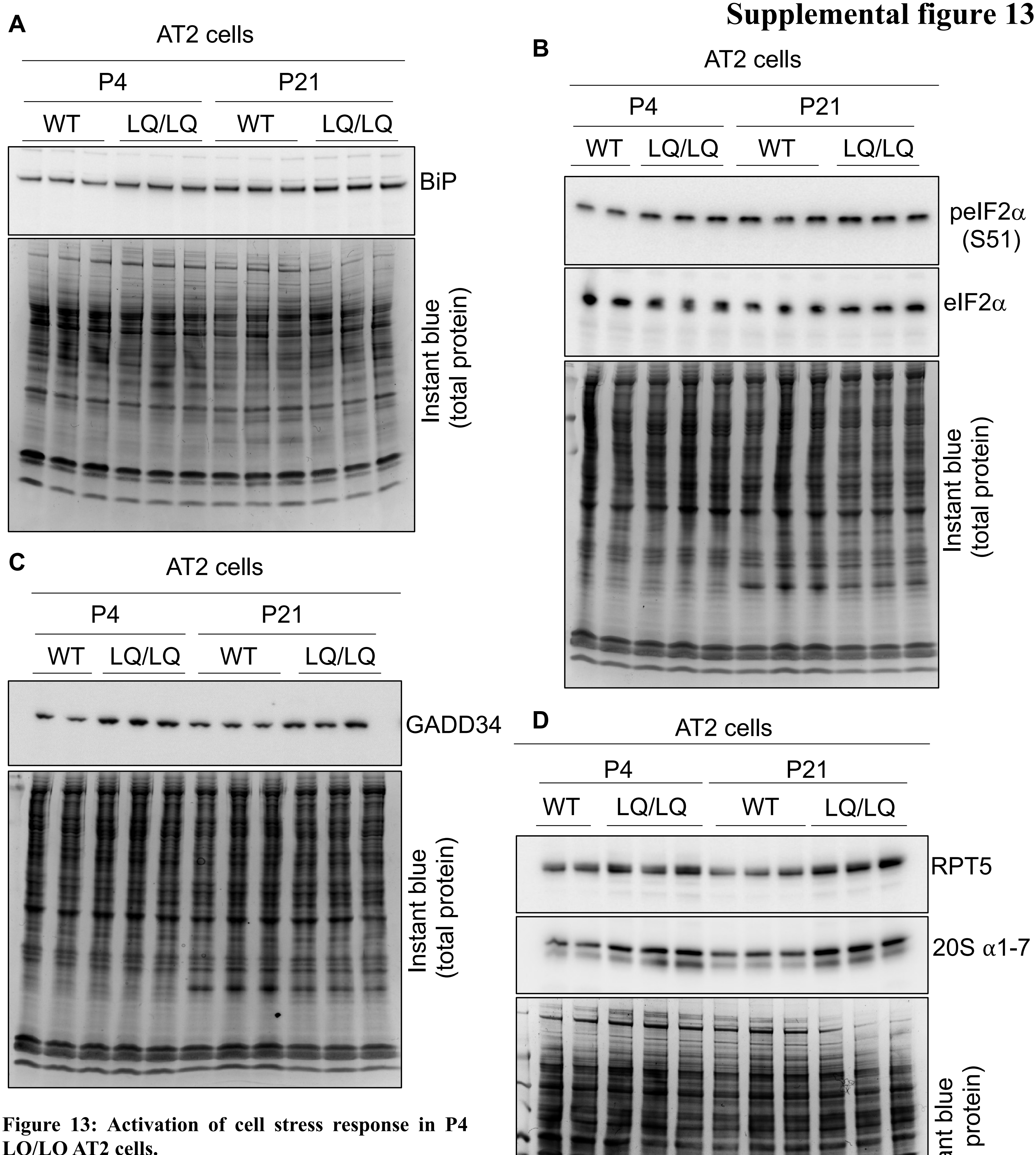

Figure 13: Activation of cell stress response in P4 LQ/LQ AT2 cells.

A-D. Western blot analyses of 20-30 μg of AT2 cell lysates separated by SDS-PAGE. Gels were stained post-transfer with Coomassie-based instant blue to assess protein loading. Data was normalized to total protein using the BioRad ImageLab software. Blot shown in figure 3 was stripped and re-probed for BiP (same gel for total protein).

Supplemental table 1: List of primary antibodies used for immunofluorescence (IF) or western blotting (WB). Rb: rabbit, GP: guinea pig, Ms: mouse, Gt: goat, Rt: rat.

Antibody	Company	Host	Catalog #	Clone	Lot	Dilution (IF)	Dilution (WB)	Antigen retrieval (IF)
α1–7	Abcam	Ms	ab22674	MCP231	GR3179905-20	-	1;1000	-
ABCA3	Seven Hills Bioreagents	Rb	70565	-	0712A	1;100	-	Sodium citrate+heat
ABCA3	In house	GP	985	-	Bl: 06/09/05	1;100	-	Sodium citrate+heat
AGER	R&D systems	Gt	AF1145	-	HCE0717082	1;1000		Sodium citrate+heat
α-smooth muscle actin (αSMA)	Sigma	Ms	A5228	1A4		1;5000	-	Sodium citrate+heat
BAX	Cell Signaling Technologies	Rb	14796S			-	1;1000	-
BiP	Sigma	Rb	G9043	ET-21	-	-	1;20000	-
CCSP	Seven Hills Bioreagents	GP	210	-	Bl: 3/10/11	1;100		Sodium citrate+heat
E-Cadherin (ECAD)	R&D systems	Rt	MAB7481	114420	JZQ0311121	1;1000	-	Sodium citrate+heat
eIF2α	Cell Signaling Technologies	Ms	2103S	L57A5	5	-	1;1000	-
GADD34	Thermo Fisher Scientific	Rb	PA1-139	-	UG286154	-	1;1000	-
HOPX	Santa Cruz	Rb	SC-30216	-	B2616	1;100	-	Sodium citrate+heat
Ki67	BD Pharmingen	Ms	556003	-	6251774	1;100	-	Sodium citrate+heat
Mature SP-C	Seven Hills Bioreagents	Rb	76694	-	-	-	1;15000	-
NKX2.1	Seven Hills Bioreagents	Ms	8G7G31	8G7G31	1211C	1;100	-	Sodium citrate+heat
NKX2.1	Seven Hills Bioreagents	GP	G237	-	Bl: 12/16/11	1;100	-	Sodium citrate+heat
peIF2α (S51)	Cell Signaling Technologies	Rb	3597S	119A11	9	-	1;1000	-
proSP-C_{NT}	Seven Hills Bioreagents	Rb	458	-	Bl: 3/23/11	1;100	-	Compatible with sodium citrate+heat
proSP-C_{CT}	In house	GP	992	-	Bl: 12/13/07	1;1000	-	Compatible with sodium citrate+heat

Antibody	Company	Host	Catalog #	Clone	Lot	Dilution (IF)	Dilution (WB)	Antigen retrieval (IF)
proSP-C_{NT}	Seven Hills Bioreagents	Rb	9337	-	364	1;1000	1;15000	Compatible with sodium citrate+heat
RPT5	Bethyl Laboratories	Rb	A303-538A	-	-	-	1;2000	-
SOX2	Seven Hills Bioreagents	Rb	1236	-	1410B	1;1000	-	Sodium citrate+heat

Supplemental table 2: List of secondary antibodies used for immunofluorescence (IF) or western blotting (WB).

Antibody	Source	Catalog number	Dilution (WB)	Dilution (IF)
Donkey anti goat Alexa 568	Invitrogen	A11057	-	1;200
Donkey anti goat Alexa 647	Invitrogen	A21447		
Donkey anti guinea pig Alexa 488	Jackson ImmunoResearch	706-545-148		
Donkey anti guinea pig Alexa 647	Jackson ImmunoResearch	706-605-148		
Donkey anti mouse Alexa 647	Invitrogen	A31571		
Donkey anti rabbit Alexa 488	Invitrogen	A21206		
Donkey anti rabbit Alexa 568	Invitrogen	A10042		
Donkey anti rabbit Alexa 594	Invitrogen	A21206		
Donkey anti rabbit Alexa 647	Invitrogen	A31573		
Donkey anti rat Alexa 594	Invitrogen	A21209		
Goat anti guinea pig Alexa 488	Invitrogen	A11073		
Goat anti guinea pig Alexa 568	Invitrogen	A11075		
Goat anti guinea pig Alexa 647	Invitrogen	A21450		
Goat anti mouse Alexa 647	Invitrogen	A21236		
Goat anti rabbit Alexa 488	Invitrogen	A11034		
Goat anti rabbit Alexa 568	Invitrogen	A11011		
Goat anti mouse HRP	EMD Millipore	401215	1:20000	-
Goat anti rabbit HRP	EMD Millipore	401315		

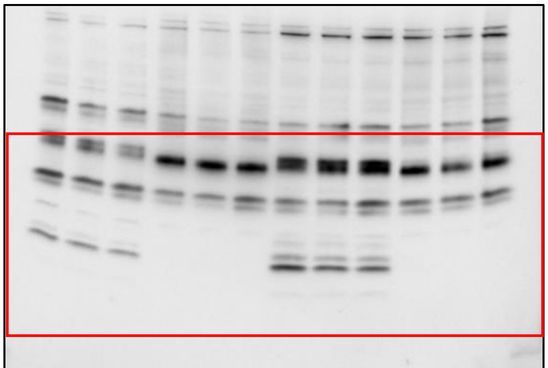

Full unedited gel for fig. 3A (proSP-C)

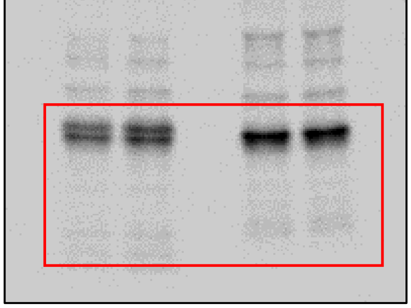

Full unedited gel for fig. 3D (proSP-C, lower exposure)

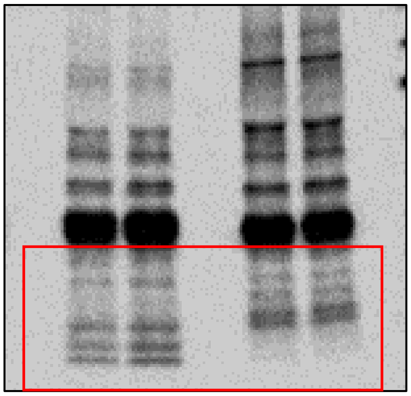

Full unedited gel for fig. 3D (proSP-C, higher exposure)

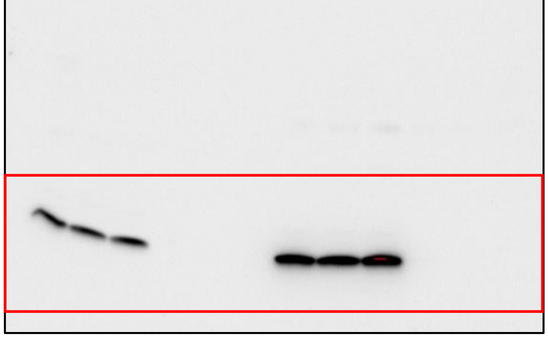

Full unedited gel for fig. 3A (mature SP-C)

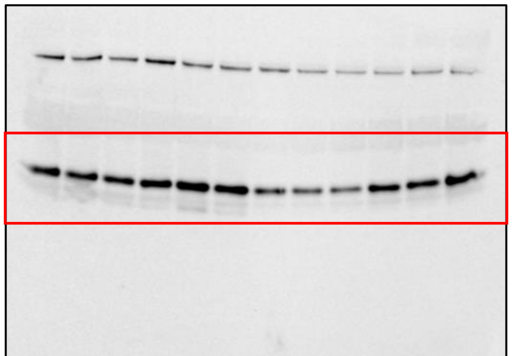

Full unedited gel for fig. 5B (BAX)

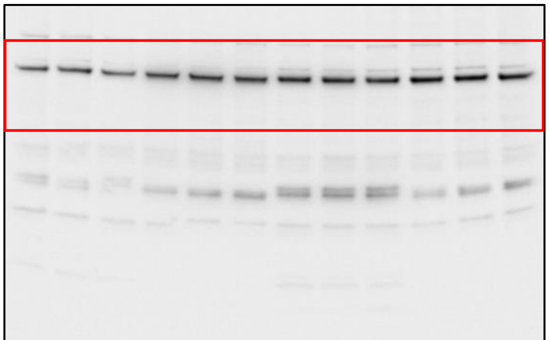

Full unedited gel for supplemental fig. 13A (BiP)

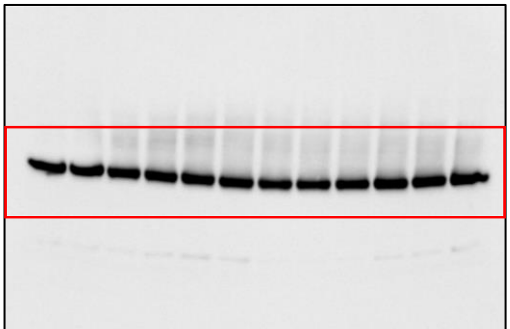

Full unedited gel for fig. 5B (Actin)

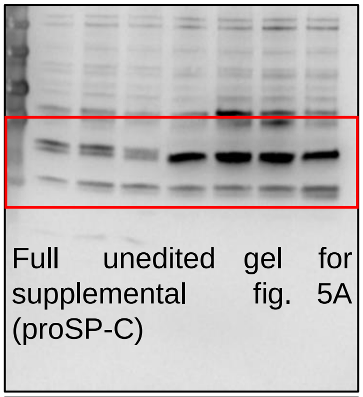

Full unedited gel for supplemental fig. 5A (proSP-C)

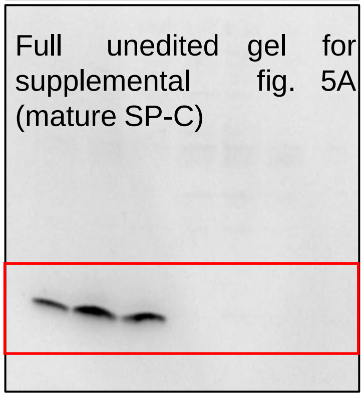

Full unedited gel for supplemental fig. 5A (mature SP-C)

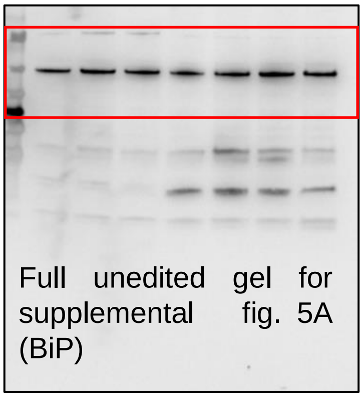

Full unedited gel for supplemental fig. 5A (BiP)

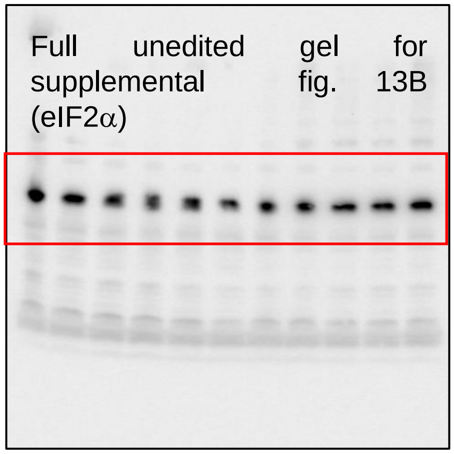

Full unedited gel for supplemental fig. 13B (eIF2α)

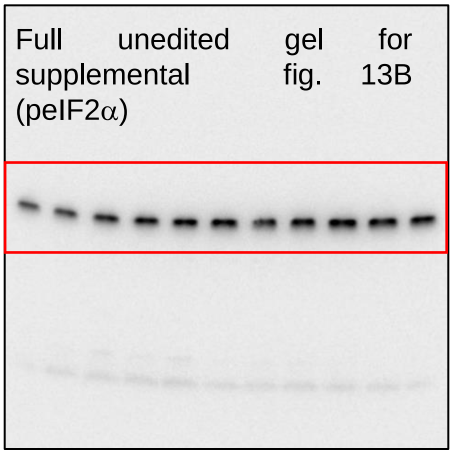

Full unedited gel for supplemental fig. 13B (peIF2α)

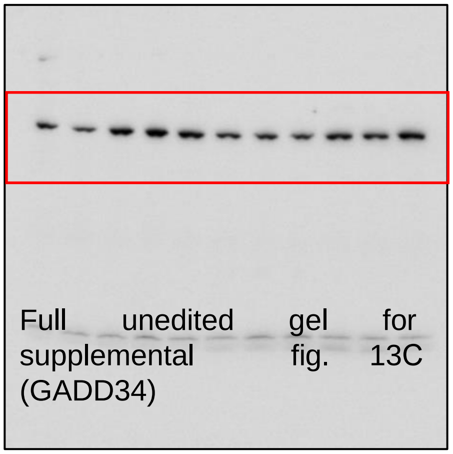

Full unedited gel for supplemental fig. 13C (GADD34)

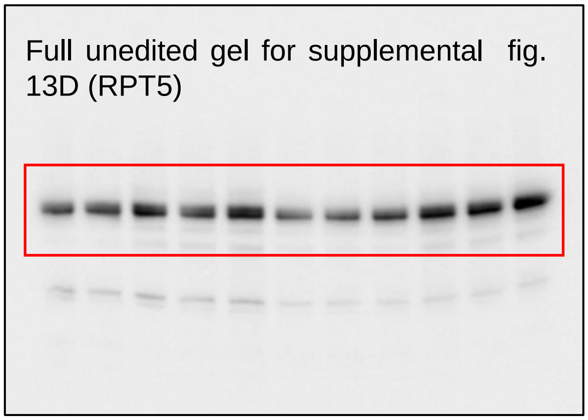

Full unedited gel for supplemental fig. 13D (RPT5)

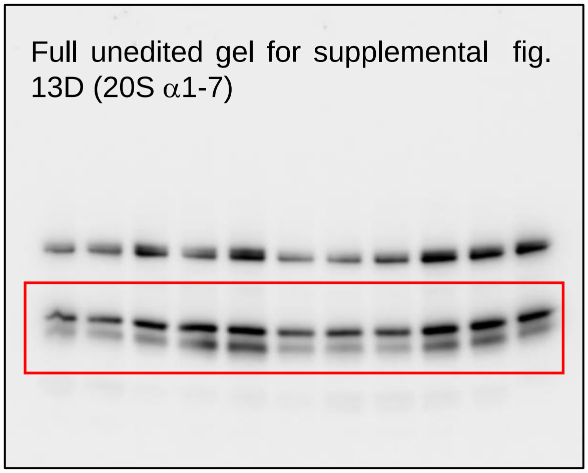

Full unedited gel for supplemental fig. 13D (20S α1-7)
